# Structural Properties of the Wyner–Ziv Rate Distortion Function: Applications for Multivariate Gaussian Sources †

**DOI:** 10.3390/e26040306

**Published:** 2024-03-29

**Authors:** Michail Gkagkos, Charalambos D. Charalambous

**Affiliations:** 1 Department of Electrical and Computer Engineering, Texas A & M University, College Station, TX 77843, USA; gkagkos@tamu.edu; 2 Department of Electrical and Computer Engineering, University of Cyprus, P.O. Box 20537, CY-1678 Nicosia, Cyprus

**Keywords:** Wyner’s side information, multivariate Gaussian sources, test channel distributions

## Abstract

The main focus of this paper is the derivation of the structural properties of the test channels of Wyner’s operational information rate distortion function (RDF), $\bar{R}\left(\Delta_{X}\right)$, for arbitrary abstract sources and, subsequently, the derivation of additional properties for a tuple of multivariate correlated, jointly independent, and identically distributed Gaussian random variables, ${\left\{X_{t},Y_{t}\right\}}_{t=1}^{\infty }$, $X_{t}:\Omega \to R^{n_{x}}$, $Y_{t}:\Omega \to R^{n_{y}}$, with average mean-square error at the decoder and the side information, ${\left\{Y_{t}\right\}}_{t=1}^{\infty }$, available only at the decoder. For the tuple of multivariate correlated Gaussian sources, we construct optimal test channel realizations which achieve the informational RDF, $\bar{R}\left(\Delta_{X}\right)\overset{▵}{=}\inf_{M(\Delta_{X})}I\left(X;Z|Y\right)$, where $M(\Delta_{X})$ is the set of auxiliary RVs *Z* such that $P_{Z|X,Y}=P_{Z|X}$, $\hat{X}=f\left(Y,Z\right)$, and $E\{||X-\hat{X}{\left|\right|}^{2}\}\leq \Delta_{X}$. We show the following fundamental structural properties: (1) Optimal test channel realizations that achieve the RDF and satisfy conditional independence, $P_{X|\hat{X},Y,Z}=P_{X|\hat{X},Y}=P_{X|\hat{X}},\;E\left\{X|\hat{X},Y,Z\right\}=E\left\{X|\hat{X}\right\}=\hat{X}.$ (2) Similarly, for the conditional RDF, $R_{X|Y}\left(\Delta_{X}\right)$, when the side information is available to both the encoder and the decoder, we show the equality $\bar{R}\left(\Delta_{X}\right)=R_{X|Y}\left(\Delta_{X}\right)$. (3) We derive the water-filling solution for $R_{X|Y}\left(\Delta_{X}\right)$.

## 1. Introduction, Problem Statement, and Main Results

### 1.1. The Wyner and Ziv Lossy Compression Problem and Generalizations

**Figure 1:**
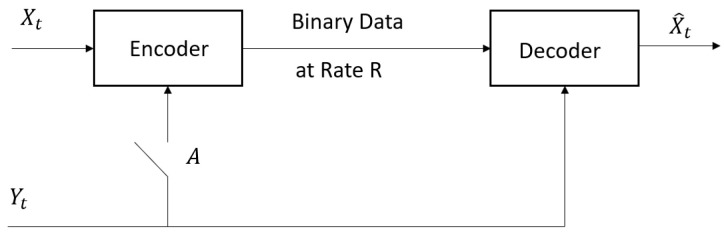
The Wyner and Ziv [1] block diagram of lossy compression. If switch A is closed, then the side information is available at both the encoder and the decoder; if switch A is open, the side information is only available at the decoder.

Wyner and Ziv [1] derived an operational information definition for the lossy compression problem in Figure 1 with respect to a single-letter fidelity of reconstruction. The joint sequence of random variables (RVs) $\left\{\left(X_{t},Y_{t}\right):t=1,2,\cdots \right\}$ takes values in sets of finite cardinality, {X,Y}, and it is generated independently according to the joint probability distribution function $P_{X,Y}$. Wyner [2] generalized [1] to RVs $\left\{\left(X_{t},Y_{t}\right):t=1,2,\cdots \right\}$ that take values in abstract alphabet spaces {X,Y} and hence include continuous-valued RVs.

*(A) Switch “A” Closed:* When the side information $\left\{Y_{t}:t=1,2,\cdots \right\}$ is available non-causally at both the encoder and the decoder, Wyner [2] (see also Berger [3]) characterized the infimum of all achievable operational rates (denoted by ${\bar{R}}_{1}\left(\Delta_{X}\right)$ in [2]), subject to a single-letter fidelity with average distortion less than or equal to $\Delta_{X}\in \left[0,\infty \right)$. The rate is given by the single-letter operational information theoretic conditional RDF:

(1) $$\begin{array}{cc} R_{X|Y}\left(\Delta_{X}\right)\overset{▵}{=} & \underset{M_{0}\left(\Delta_{X}\right)}{\inf}I\left(X;\hat{X}|Y\right)\in \left[0,\infty \right],\;\Delta_{X}\in \left[0,\infty \right) \end{array}$$

(2) $$\begin{array}{cc} = & \underset{P_{\hat{X}|X,Y}:E\left\{d_{X}\left(X,\hat{X}\right)\right\}\leq \Delta_{X}}{\inf}I\left(X;\hat{X}|Y\right) \end{array}$$

where $M_{0}\left(\Delta_{X}\right)$ is the set specified by

(3) $$\begin{array}{cc} M_{0}\left(\Delta_{X}\right)\overset{▵}{=} & \{\hat{X}:\Omega \to \hat{X}:\;P_{X,Y,\hat{X}}\;\;\mathrm{is}\;\mathrm{the}\;\mathrm{joint}\;\mathrm{measure}\;\mathrm{on}X\times Y\times \hat{X},\\ \; & E\left\{d_{X}\left(X,\hat{X}\right)\right\}\leq \Delta_{X}\}, \end{array}$$

and $\hat{X}$ is the reproduction of *X*. $I(X;\hat{X}|Y)$ is the conditional mutual information between *X* and $\hat{X}$ conditioned on *Y*, and $d_{X}\left(\cdot ,\cdot \right)$ is the fidelity criterion between *x* and $\hat{x}$. The infimum in (1) is over all elements of $M_{0}\left(\Delta_{X}\right)$ with induced joint distributions $P_{X,Y,\hat{X}}$ of the RVs $\left(X,Y,\hat{X}\right)$ such that the marginal distribution $P_{X,Y}$ is the fixed joint distribution of the source (X,Y). This problem is equivalent to (2) [4].

*(B) Switch “A” Open:* When the side information is available non-causally only at the decoder, Wyner [2] characterized the infimum of all achievable operational rates (denoted by $R^{*}\left(\Delta_{X}\right)$ in [2]), subject to a single-letter fidelity with average distortion less than or equal to $\Delta_{X}$. The rate is given by the single-letter operational information theoretic RDF as a function of an auxiliary RV Z:Ω→Z:

(4) $$\begin{array}{cc} \bar{R}\left(\Delta_{X}\right)\overset{▵}{=} & \underset{M(\Delta_{X})}{\inf}\left\{I(X;Z)-I(Y;Z)\right\}\in \left[0,\infty \right],\;\Delta_{X}\in \left[0,\infty \right) \end{array}$$

(5) $$\begin{array}{cc} = & \underset{M(\Delta_{X})}{\inf}I\left(X;Z|Y\right) \end{array}$$

where $M(\Delta_{X})$ is specified by the set of auxiliary RVs *Z* and defined as:

(6) $$\begin{array}{cc} \; & M\left(\Delta_{X}\right)\overset{▵}{=}\{Z:\Omega \to Z:\;P_{X,Y,Z,\hat{X}}\;\mathrm{is}\;\mathrm{the}\;\mathrm{joint}\;\mathrm{measure}\;\mathrm{on}X\times Y\times Z\times \hat{X},\\ \; & P_{Z|X,Y}=P_{Z|X},\;\exists \;\mathrm{meas}.\mathrm{fun}.f:Y\times Z\to \hat{X},\;\hat{X}=f\left(Y,Z\right),\;E\left\{d_{X}\left(X,\hat{X}\right)\right\}\leq \Delta_{X}\}. \end{array}$$

**Figure 2:**
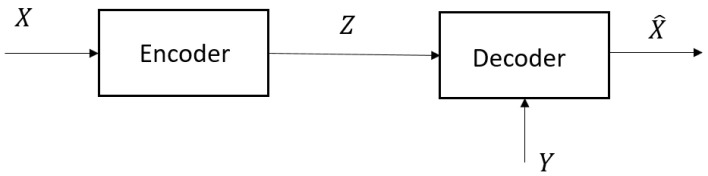
Test channel when side information is only available to the decoder.

Wyner’s realization of the joint measure $P_{X,Y,Z,\hat{X}}$ induced by the RVs $\left(X,Y,Z,\hat{X}\right)$ is illustrated in Figure 2, where *Z* is the output of the “test channel”, $P_{Z|X}$. Clearly, $\bar{R}\left(\Delta_{X}\right)$ involves two strategies, i.e., f(·,·) and $P_{Z|X,Y}=P_{Z|X}$. This makes it a much more complex problem compared to $R_{X|Y}\left(\Delta_{X}\right)$ (which involves only $P_{\hat{X}|X,Y}$).

Throughout [2], the following assumption is imposed.

**Assumption 1.**  *I(X;Y)<∞ (see [2]).*

Wyner [2] considered scalar-valued jointly Gaussian RVs (X,Y) with square-error distortion and constructed the optimal realizations $\hat{X}$ and $\left(Z,\hat{X}\right)$ and the function f(X,Z) from the sets $M_{0}\left(\Delta_{X}\right)$ and $M(\Delta_{X})$, respectively. Also, it is shown that these realizations achieve the characterizations of the RDFs $R_{X|Y}\left(\Delta_{X}\right)$ and $\bar{R}\left(\Delta_{X}\right)$, respectively, and that the two rates are equal, i.e., $\bar{R}\left(\Delta_{X}\right)=R_{X|Y}\left(\Delta_{X}\right)$.

*(C) Marginal RDF:* If there is no side information, $\left\{Y_{t}:t=1,2,\ldots \right\}$, or the side information is independent of the source, $\left\{X_{t}:t=1,2,\ldots \right\}$, the RDFs $R_{X|Y}\left(\Delta_{X}\right)$ and $\bar{R}\left(\Delta_{X}\right)$ degenerate to the marginal RDF $R_{X}\left(\Delta_{X}\right)$, defined by

(7) $$\begin{array}{c} R_{X}\left(\Delta_{X}\right)\overset{▵}{=}\underset{P_{\hat{X}|X}:E\left\{d_{X}\left(X,\hat{X}\right)\right\}\leq \Delta_{X}}{\inf}I\left(X;\hat{X}\right)\in \left[0,\infty \right],\;\Delta_{X}\in \left[0,\infty \right). \end{array}$$

*(D) Gray’s Lower Bounds:* A lower bound on $R_{X|Y}\left(\Delta_{X}\right)$ is given by Gray in [4] [Theorem 3.1]. This bound connects $R_{X|Y}\left(\Delta_{X}\right)$ with the marginal RDF and the mutual information between *X* and *Y* as follows:

(8) $$\begin{array}{c} R_{X|Y}\left(\Delta_{X}\right)\geq R_{X}\left(\Delta_{X}\right)-I\left(X;Y\right). \end{array}$$

Clearly, the lower bound is trivial for values of $\Delta_{X}\in \left[0,\infty \right)$ such that $R_{X}\left(\Delta_{X}\right)-I\left(X;Y\right)<0$.

### 1.2. Main Contributions of the Paper

We first consider Wyner’s [2] RDFs $R_{X|Y}\left(\Delta_{X}\right)$ and $\bar{R}\left(\Delta_{X}\right)$ for arbitrary RVs (X,Y) defined on abstract alphabet spaces, and we derive structural properties of the realizations that achieve the two optimal test channels. Subsequently, we generalize Wyner’s [2] results to multivariate-valued jointly Gaussian RVs (X,Y). In other words, we construct the optimal multivariate-valued realizations $\hat{X}$ and $\left(\hat{X},Z\right)$ and the function f(X,Z) which achieve the RDFs $R_{X|Y}\left(\Delta_{X}\right)$ and $\bar{R}\left(\Delta_{X}\right)$, respectively. In the literature, it is often called achievability of the converse coding theorem. In addition, we use the realizations to prove the equality $\bar{R}\left(\Delta_{X}\right)=R_{X|Y}\left(\Delta_{X}\right)$ and to derive the water-filling solution. Along the way, we verify that our results reproduce, for scalar-valued RVs (X,Y), Wyner [2] RDFs and the optimal realizations. However, to our surprise, the existing results from the literature [[5], Theorem 4 and Abstract and [6], Theorem 3A], which deal with the more general multivariate-valued remote sensor problem (the RDF of the remote sensor problem is a generalization of Wyner’s RDF $\bar{R}\left(\Delta_{X}\right)$, with the encoder observing a noisy version of the RVs generated by the source), do not degenerate to Wyner’s [2] RDFs, when specilized to scalar-valued RVs (we verify this in Remark 5 by also checking the correction suggested in https://tiangroup.engr.tamu.edu/publications/) (accessed on 3 January 2024). In Section 1.3, we give a detailed account of the main results of this paper. We should emphasize that preliminary results of this paper appeared in [7], mostly without the details of the proofs. This paper is extended [7] and contains complete proofs of the preliminary results of [7], which in some cases are lengthy (see, for example, Section 4, proofs of Theorems 3–5, Corollaries 1 and 2, etc.).

### 1.3. Problem Statement and Main Results

**(a)** We consider a tuple of jointly independent and identically distributed (i.i.d.) arbitrary RVs $\left(X^{n},Y^{n}\right)=\left\{\left(X_{t},Y_{t}\right):t=1,2,\ldots ,n\right\}$ defined on abstract alphabet spaces, and we derive the following results.

**(a.1) Lemma 1:** Achievable lower bound on the conditional mutual information $I(X;\hat{X}|Y)$, which strengthens Gray’s lower bound (8) [[4], Theorem 3.1].

**(a.2) Theorem 2:** Structural properties of the optimal reconstruction $\hat{X}$, which achieves a lower bound on $R_{X|Y}\left(\Delta_{X}\right)$ for mean-square error distortion. Theorem 2 strengthens the conditions for the equality to hold, $R_{X|Y}\left(\Delta_{X}\right)=\bar{R}\left(\Delta_{X}\right)$, given by Wyner [2] [Remarks, p. 65] (see Remark 1). However, for finite-alphabet-valued sources with Hamming distance distortion, it might be the case that $R_{X|Y}\left(\Delta_{X}\right)<\bar{R}\left(\Delta_{X}\right)$, as pointed out by Wyner and Ziv [1] [Section 3] for the doubly symmetric binary source.

**(b)** We consider a tuple of jointly i.i.d. multivariate Gaussian RVs $\left(X^{n},Y^{n}\right)=\left\{\left(X_{t},Y_{t}\right):t=1,2,\ldots ,n\right\}$, with respect to the square-error fidelity, as defined below.

(9) $$\begin{array}{cc} \; & X_{t}:\Omega \to R^{n_{x}}=X,\;Y_{t}:\Omega \to R^{n_{y}}=Y,\;t=1,2,\ldots ,n, \end{array}$$

(10) $$\begin{array}{cc} \; & X_{t}\in N\left(0,Q_{X}\right),\;Y_{t}\in N\left(0,Q_{Y}\right), \end{array}$$

(11) $$\begin{array}{cc} \; & Q_{\left(X_{t},Y_{t}\right)}=E\left\{\left[\begin{array}{c} X_{t}\\ Y_{t} \end{array}\right]{\left[\begin{array}{c} X_{t}\\ Y_{t} \end{array}\right]}^{T}\right\}=\left[\begin{array}{cc} Q_{X} & Q_{X,Y}\\ Q_{X,Y}^{T} & Q_{Y} \end{array}\right], \end{array}$$

(12) $$\begin{array}{cc} \; & P_{X_{t},Y_{t}}=P_{X,Y}\;\mathrm{multivariate}\;\mathrm{Gaussian}\;\mathrm{distribution}, \end{array}$$

(13) $$\begin{array}{cc} \; & {\hat{X}}_{t}:\Omega \to R^{n_{x}}=X,\;t=1,2\ldots ,n, \end{array}$$

(14) $$\begin{array}{cc} \; & D_{X}\left(x^{n},{\hat{x}}^{n}\right)=\frac{1}{n}\sum_{t=1}^{n}\left|\right|x_{t}-{\hat{x}}_{t}{\left|\right|}_{R^{n_{x}}}^{2}, \end{array}$$

where $n_{x},n_{y}$ are arbitrary positive integers, $X\in N(0,Q_{X})$ means *X* is a Gaussian RV, with zero mean and covariance matrix $Q_{X}$, and $\left|\right|\cdot {\left|\right|}_{R^{n_{x}}}^{2}$ is the Euclidean distance on $R^{n_{x}}$. To give additional insight we often consider the following realization of side information (the condition $DD^{T}≻0$ ensures I(X;Y)<∞, and hence, Assumption 1 is respected).

(15) $$\begin{array}{cc} \; & Y_{t}=CX_{t}+DV_{t}, \end{array}$$

(16) $$\begin{array}{cc} \; & V_{t}\in N\left(0,Q_{V}\right), \end{array}$$

(17) $$\begin{array}{cc} \; & C\in R^{n_{y}\times n_{x}},\;D\in R^{n_{y}\times n_{y}},\;DD^{T}≻0,\;Q_{V}=I_{n_{y}}, \end{array}$$

(18) $$\begin{array}{cc} \; & V^{n}\;\mathrm{independent}\;\mathrm{of}X^{n}, \end{array}$$

where $I_{n_{y}}$ denotes the $n_{y}\times n_{y}$ identity matrix. For the above specification of the source and distortion criterion, we derive the following results.

**(b.1) Theorems 3 and 4:** Structural properties of optimal realization of $\hat{X}$, which achieves $R_{X|Y}\left(\Delta_{X}\right)$, its closed form expression.

**(b.2) Theorem 5:** Structural properties of optimal realization of $\hat{X}$ and $\hat{X}=f\left(Y,Z\right)$, which achieve $\bar{R}\left(\Delta_{X}\right)$ and the closed form expression of $\bar{R}\left(\Delta_{X}\right)$.

**(b.3) A proof that** $\bar{R}\left(\Delta_{X}\right)$ **and** $R_{X|Y}\left(\Delta_{X}\right)$ **coincide:** Calculation of the distortion region such that Gray’s lower bound (8) holds with equality.

In Remark 4, we consider the tuple of scalar-valued, jointly Gaussian RVs (X,Y) with square error distortion function and verify that our optimal realizations of $\hat{X}$ and the closed form expressions for $R_{X|Y}\left(\Delta_{X}\right)$ and $\bar{R}\left(\Delta_{X}\right)$ are identical to Wyner’s [2] realizations and RDFs.

We should emphasize that our methodology is different from past studies in the sense that we focus on the structural properties of the realizations of the test channels, that achieve the characterizations of the two RDFs (i.e., verification of the converse coding theorem). Our derivations are generic and bring new insight into the construction of realizations that induce the optimal test channels of other distributed source coding problems (i.e., establishing the achievability of the converse coding theorem).

### 1.4. Additional Generalizations of the Wyner-Ziv [1] and Wyner [2] RDFs

Below, we discuss additional generalizations of Wyner and Ziv [1] and Wyner’s [2] RDFs.

*(A) Draper and Wornell [8] Distributed Remote Source Coding Problem:* Draper and Wornell [8] generalized the RDF $\bar{R}\left(\Delta_{X}\right)$, when the source to be estimated at the decoder is S:Ω→S, and it is not directly observed at the encoder. Rather, the encoder observes a RV X:Ω→X (which is correlated with *S*), while the decoder observes another RV, as side information, Y:Ω→Y, which provides information on (S,X). The aim is to reconstruct *S* at the decoder by $\hat{S}:\Omega \to \hat{S}$, subject to an average distortion $E\left\{d_{S}\left(S,\hat{S}\right)\right\}\leq \Delta_{S}$, by a function $\hat{S}=f\left(Y,Z\right)$. The RDF for this problem, called the distributed remote source coding problem, is defined by [8]

(19) $$\begin{array}{cc} {\bar{R}}^{PO}\left(\Delta_{S}\right)= & \underset{M^{PO}\left(\Delta_{S}\right)}{\inf}I\left(X;Z|Y\right)\in \left[0,\infty \right], \end{array}$$

where $M^{PO}\left(\Delta_{S}\right)$ is specified by the set of auxiliary RVs *Z*, and defined as:

(20) $$\begin{array}{cc} M^{PO}\left(\Delta_{S}\right)\overset{▵}{=} & \{Z:\Omega \to Z:\;P_{S,X,Y,Z,\hat{X}}\;\;\mathrm{is}\;\mathrm{the}\;\mathrm{joint}\;\mathrm{measure}\;\mathrm{on}S\times X\times Y\times Z\times \hat{X},\\ \; & P_{Z|S,X,Y}=P_{Z|X},\exists \;\mathrm{measurable}\;\mathrm{function}\;f^{PO}:Y\times Z\to \hat{S},\\ \; & \hat{S}=f^{PO}\left(Y,Z\right),\;E\left\{d_{S}\left(S,\hat{S}\right)\right\}\leq \Delta_{S}\}. \end{array}$$

Clearly, if S=X−a.s (almost surely), then ${\bar{R}}^{PO}\left(\Delta_{S}\right)$ degenerates (this implies the optimal test channel that achieves the characterization of the RDF ${\bar{R}}^{PO}\left(\Delta_{S}\right)$ should degenerate to the optimal test channel that achieves the characterization of the RDF $\bar{R}\left(\Delta_{X}\right)$) to $\bar{R}\left(\Delta_{X}\right)$. For scalar-valued jointly Gaussian RVs $\left(S,X,Y,Z,\hat{X}\right)$ with square-error distortion, Draper and Wornell [8] [Equation (3) and Appendix A.1] derived the characterization of the RDF ${\bar{R}}^{PO}\left(\Delta_{S}\right)$ and constructed the optimal realization $\hat{S}=f^{PO}\left(Y,Z\right)$, which achieves this characterization.

In [5,6], the authors investigated the RDF ${\bar{R}}^{PO}\left(\Delta_{S}\right)$ of [8] for the multivariate jointly Gaussian RVs $\left(S,X,Y,Z,\hat{X}\right)$, with square-error distortion, and derived a characterization for the RDF ${\bar{R}}^{PO}\left(\Delta_{S}\right)$ in [[5], Theorem 4] and [[6], Theorem 3A] (see [[6], Equation (26)]). However, it will become apparent in Remark 5 that, when S=X− almost surely (a.s.), and hence ${\bar{R}}^{PO}\left(\Delta_{S}\right)=\bar{R}\left(\Delta_{X}\right)$, the RDFs given in [[5], Theorem 4] and [[6], Theorem 3A], do not produce Wyner’s [2] value. We also show in Remark 5 that the same technical issues occur for the correction suggested in https://tiangroup.engr.tamu.edu/publications/ (accessed on 3 January 2024). Similarly, when S=X−a.s. and Y=X−a.s. [[5], Theorem 4] and [[6], Theorem 3A], do not produce the classical RDF $R_{X}\left(\Delta_{X}\right)$ of the Gaussian source *X*.

*(B) Additional Literature Review:* The formulation of Figure 1 is generalized to other multiterminal or distributed lossy compression problems, such as relay networks, sensor networks, etc., under various code formulations and assumptions. Oohama [9] analyzed lossy compression problems for a tuple of scalar correlated Gaussian memoryless sources with square error distortion criterion. Also, he determined the rate-distortion region, in the special case when one source provides partial side information to the other source. Furthermore, Oohama in [10] analyzed separate lossy compression problems for L+1 scalar correlated Gaussian memoryless sources, when *L* of the sources provide partial side information at the decoder for the reconstruction of the remaining source and gave a partial answer to the rate distortion region. Additionally, ref. [10] proved that the problem of [10] includes, as a special case, the additive white Gaussian CEO problem analyzed by Viswanathan and Berger [11]. Extensions of [10] are derived by Ekrem and Ulukus [12] and Wang and Chen [13], where an outer bound on the rate region is derived for the vector Gaussian multiterminal source. Additional works are [14,15,16] and the references therein.

The vast literature on multiterminal or distributed lossy compression of jointly Gaussian sources with square-error distortion (including the references mentioned above), is often confined to scalar-valued correlated RVs. Moreover, as easily verified, not much emphasis is given in the literature on the structural properties of the realizations of RVs that induce the optimal test channels that achieve the characterizations of the RDFs.

The rest of the paper is organized as follows. In Section 2, we review Wyner’s [2] operational definition of lossy compression. We also state a fundamental theorem on mean-square estimation that we use throughout the paper regarding the analysis of **(b)**. The main Theorems are presented in Section 3; some of the proofs, including the structural properties, are given in Section 4. Connections between our results and the past literature are provided in Section 5. A simulation to show the gap between the two rates is given in the same section.

## 2. Preliminaries

In this section, we review the Wyner [2] source coding problems with fidelity in Figure 1. We begin with the notation, which follows closely [2].

### 2.1. Notation

Let $Z\overset{▵}{=}\left\{\ldots ,-1,0,1,\ldots \right\}$ the set of all integers, $N\overset{▵}{=}\left\{0,1,2,\ldots ,\right\}$ the set of natural integers, $Z_{+}\overset{▵}{=}\left\{1,2,\ldots ,\right\}$. For $n\in Z_{+}$, denote the following finite subset of the above defined set, $Z_{n}\overset{▵}{=}\left\{1,2,\ldots ,n\right\}$. Denote the real numbers by R and the set of positive and of strictly positive real numbers, by $R_{+}=\left[0,\infty \right)$ and $R_{++}=\left(0,\infty \right)$, respectively.

For any matrix $A\in R^{p\times m},\left(p,m\right)\in Z_{+}\times Z_{+}$, we denote its kernel by ker(A) its transpose by $A^{T}$, and for m=p, we denote its trace by trace(A), and by diag{A}, the matrix with diagonal entries $A_{ii},\;i\in Z_{p}$, and zero elsewhere. The determinant of a square matrix *A* is denoted by det(A). The identity matrix with dimensions p×p is designated as $I_{p}$. Denote an arbitrary set or space by U and the product space formed by *n* copies of it by $U^{n}\overset{▵}{=}\times_{t=1}^{n}U$. $u^{n}\in U^{n}$ denotes the set of n−tuples $u^{n}\overset{▵}{=}\left(u_{1},u_{2},\ldots ,u_{n}\right)$, where $u_{k}\in U,k\in Z_{k}$ are its coordinates. Denote a probability space by (Ω,F,P). For a sub-sigma-field G⊆F, and A∈F, denote by P(A|G) the conditional probability of *A* given G; i.e., P(A|G)=P(A|G)(ω),ω∈Ω is a measurable function on Ω.

On the above probability space, consider two-real valued random variables (RV) X:Ω→X,Y:Ω→X, where (X,B(X)),(Y,B(Y)) are arbitrary measurable spaces. The measure (or joint distribution if X,Y are Euclidean spaces) induced by (X,Y) on X×Y is denoted by $P_{X,Y}$ or P(dx,dy) and their marginals on X and Y by $P_{X}$ and $P_{Y}$, respectively. The conditional measure of RV *X* conditioned on *Y* is denoted by $P_{X|Y}$ or P(dx|y), when Y=y is fixed. On the above probability space, consider three-real values RVs X:Ω→X,Y:Ω→X, Z:Ω→Z. We say that RVs (Y,Z) are conditional independent given RV *X* if $P_{Y,Z|X}=P_{Y|X}P_{Z|X}-a.s.$ (almost surely) or equivalently $P_{Z|X,Y}=P_{Z|X}-a.s$; the specification a.s is often omitted. We often denote the above conditional independence by the Markov chain (MC) Y↔X↔Z.

Finally, for RVs X,Y, etc., H(X) denotes differential entropy of *X*, H(X|Y) conditional differential entropy of *X* given *Y*, and I(X;Y) the mutual information between *X* and *Y*, as defined in standard books on information theory [17,18]. We use log(·) to denote the natural logarithm. The notation $X\in N(0,Q_{X})$ means *X* is a Gaussian distributed RV with zero mean and covariance $Q_{X}⪰0$, where $Q_{X}⪰0$ (resp. $Q_{X}≻0$) means $Q_{X}$ is positive semidefinite (respectively, positive definite). We denote the covariance of *X* and *Y* by

(21) $$\begin{array}{c} Q_{X,Y}\overset{▵}{=}\mathrm{cov}\left(X,Y\right). \end{array}$$

We denote the covariance of *X* conditioned on *Y* by

(22) $$\begin{array}{cc} Q_{X|Y} & \overset{▵}{=}\mathrm{cov}\left(X,X|Y\right)\\ \; & =E\left\{\left(X-E\left(X|Y\right)\right){\left(X-E\left(X|Y\right)\right)}^{T}\right\}\;\mathrm{if}\;(X,Y)\;\mathrm{is}\;\mathrm{jointly}\;\mathrm{Gaussian}, \end{array}$$

where the second equality is due to a property of jointly Gaussian RVs.

### 2.2. Mean-Square Estimation of Conditionally Gaussian RVs

Below, we state a well-known property of conditionally Gaussian RVs from [19], which we use in our derivations.

**Proposition 1.**  *Conditionally Gaussian RVs [19]. Consider a pair of multivariate RVs $X={\left(X_{1},\ldots ,X_{n_{x}}\right)}^{T}:\Omega \to R^{n_{x}}$ and $Y={\left(Y_{1},\ldots ,Y_{n_{y}}\right)}^{T}:\Omega \to R^{n_{y}}$, $\left(n_{x},n_{y}\right)\in Z_{+}\times Z_{+}$, defined on some probability distribution $\left(\Omega ,F,P\right)$. Let G⊆F be a sub−σ−algebra. Assume the conditional distribution of (X,Y) conditioned on G, i.e., P(dx,dy|G) is P−a.s. (almost surely) Gaussian, with conditional means*

(23) $$\begin{array}{c} \mu_{X|G}\overset{▵}{=}E\left\{X|G\right\},\;\mu_{Y|G}\overset{▵}{=}E\left\{Y|G\right\}, \end{array}$$

*and conditional covariances*

(24) $$\begin{array}{c} Q_{X|G}\overset{▵}{=}\mathrm{cov}\left(X,X|G\right),\;Q_{Y|G}\overset{▵}{=}\mathrm{cov}\left(Y,Y|G\right), \end{array}$$

(25) $$\begin{array}{c} Q_{X,Y|G}\overset{▵}{=}\mathrm{cov}\left(X,Y|G\right). \end{array}$$

*Then, the vectors of conditional expectations $\mu_{X|Y,G}\overset{▵}{=}E\left\{X|Y,G\right\}$ and matrices of conditional covariances $Q_{X|Y,G}\overset{▵}{=}\mathrm{cov}\left(X,X|Y,G\right)$ are given, P−a.s., by the following expressions (If $Q_{Y|G}≻0$ then the inverse exists and the pseudoinverse is $Q_{Y|G}^{†}=Q_{Y|G}^{-1}$):*

(26) $$\begin{array}{cc} \; & \mu_{X|Y,G}=\mu_{X|G}+Q_{X,Y|G}Q_{Y|G}^{†}\left(Y-\mu_{Y|G}\right), \end{array}$$

(27) $$\begin{array}{cc} \; & Q_{X|Y,G}\overset{▵}{=}Q_{X|G}-Q_{X,Y|G}Q_{Y|G}^{†}Q_{X,Y|G}^{T}. \end{array}$$

*If G is the trivial information, i.e., G={Ω,∅}, then G is removed from the above expressions.*

Note that, if G={Ω,∅}, then (26) and (27) reduce to the well-known conditional mean and conditional covariance of *X* conditioned on *Y*.

For Gaussian RVs, we make use of the following properties.

**Proposition 2.**  *Let $X:\Omega \to R^{n},n\in Z_{+}$, $X\in N\left(0,Q_{X}\right),Q_{X}⪰0$, $S\in R^{n_{1}\times n},n_{1}\in Z_{+}$, and denote by $F^{X}$ and $F^{SX}$ the σ−algebra generated by the RVs X and SX, respectively. The following hold.*

*(a) $F^{SX}\subseteq F^{X}$.*

*(b) $F^{SX}=F^{X}$ if and only if $ker\left(Q_{X}\right)=ker\left(SQ_{X}\right)$.*

**Proof.**  This is well-known in measure theory, see [20]. □

**Proposition 3.**  *Let $X:\Omega \to R^{n},n\in Z_{+}$, $X\in N\left(0,Q_{X}\right),Q_{X}⪰0$, $\mathrm{rank}\left(Q_{X}\right)=n_{1},n_{1}\in Z_{+},n_{1}<n$. Then, there exists a linear transformation $S\in R^{n_{1}\times n}$ such that, if $X_{1}:\Omega \to R^{n_{1}}$, $X_{1}=SX$, then $X_{1}\in N\left(0,Q_{X_{1}}\right),Q_{X_{1}}≻0$, $F^{X}=F^{X_{1}}$.*

**Proof.**  This is well-known in probability theory, see [20]. □

### 2.3. Wyner’s Coding Theorems with Side Information at the Decoder

For the sake of completeness, we introduce certain results from Wyner’s work in [2], which we use in this paper. On a probability space (Ω,F,P), consider a tuple of jointly i.i.d. RVs $\left(X^{n},Y^{n}\right)=\left\{\left(Y_{t},Y_{t}\right):t\in Z_{n}\right\}$,

(28) $$\begin{array}{c} X_{t}:\Omega \to Y,\;Y_{t}:\Omega \to Y,\;\;t\in Z_{n}, \end{array}$$

with induced distribution $P_{X_{t},Y_{t}}=P_{X,Y},\forall t$. Consider also the measurable function $d_{X}:X\times \hat{X}\to \left[0,\infty \right)$, for a measurable space $\hat{X}$. Let

(29) $$\begin{array}{c} I_{M}\overset{▵}{=}\left\{0,1,\ldots ,M-1\right\},\;M\in Z_{M}, \end{array}$$

be a finite set.

A code $\left(n,M,D_{X}\right)$, when switch “A” is open (see Figure 1), is defined by two measurable functions, the encoder $F_{E}$ and the decoder $F_{D}$, with average distortion, as follows.

(30) $$\begin{array}{cc} \; & F_{E}:X^{n}⟶I_{M},\;F_{D}:I_{M}\times Y^{n}⟶{\hat{X}}^{n}, \end{array}$$

(31) $$\begin{array}{cc} \; & \frac{1}{n}E\left\{\sum_{t=1}^{n}d_{X}\left(X_{t},{\hat{X}}_{t}\right)\right\}=D_{X}, \end{array}$$

where ${\hat{X}}^{n}$ is again a sequence of RVs, ${\hat{X}}^{n}=F_{D}\left(F_{E}\left(X^{n}\right),Y^{n}\right)\in {\hat{X}}^{n}$. A non-negative rate distortion pair $\left(R,\Delta_{X}\right)$ is said to be *achievable* if for every ϵ>0, and *n* sufficiently large, there exists a code $\left(n,M,D_{X}\right)$ such that

(32) $$M\leq 2^{n(R+\epsilon )},\;D_{X}\leq \Delta_{X}+\epsilon .$$

Let R denote the set of all achievable pairs $\left(R,\Delta_{X}\right)$, and define, for $\Delta_{X}\geq 0$, the infimum of all achievable rates by

(33) $$R^{*}\left(\Delta_{X}\right)=\underset{(R,\Delta_{X})\in R}{\inf}R.$$

If for some $\Delta_{X}$ there is no R<∞ such that $(R,\Delta_{X})\in R$, then set $R^{*}\left(\Delta_{X}\right)=\infty .$ For arbitrary abstract spaces Wyner [2] characterized the infimum of all achievable rates $R^{*}\left(\Delta_{X}\right)$ by the single-letter RDF, $\bar{R}\left(\Delta_{X}\right)$ given by (5) and (6), in terms of an auxiliary RV Z:Ω→Z. Wyner’s realization of the joint measure $P_{X,Y,Z,\hat{X}}$ induced by the RVs $\left(X,Y,Z,\hat{X}\right)$ is illustrated in Figure 2, where *Z* is the output of the “test channel”, $P_{Z|X}$. Wyner proved the following coding theorems.

**Theorem 1.**  *Wyner [[2], Theorems, pp. 64–65]. Suppose Assumption 1 holds.*

*(a) Converse Theorem. For any $\Delta_{X}\geq 0$, $R^{*}\left(\Delta_{X}\right)\geq \bar{R}\left(\Delta_{X}\right)$.*

*(b) Direct Theorem. If the conditions stated in ([2], pages 64-65, (i), (ii)) hold, then $R^{*}\left(\Delta_{X}\right)\leq \bar{R}\left(\Delta_{X}\right)$, $0\leq \Delta_{X}<\infty$.*

In Figure 1, when switch *A* is closed and the tuple of jointly independent and identically distributed RVs $\left(X^{n},Y^{n}\right)$ is defined as in Section 2.3, Wyner [2] generalized Berger’s [3] characterization of all achievable pairs $\left(R,\Delta_{X}\right)$, from finite alphabet spaces to abstract alphabet spaces.

A code $\left(n,M,D_{X}\right)$, when switch “A” is closed, (see Figure 1), is defined as in Section 2.3, with the encoder $F_{E}$, replaced by

(34) $$\begin{array}{c} F_{E}:X^{n}\times Y^{n}⟶I_{M}. \end{array}$$

Let $R_{1}$ denote the set of all achievable pairs $\left(R,\Delta_{X}\right)$, again as defined in Section 2.3. For $\Delta_{X}\geq 0$, define the infimum of all achievable rates by

(35) $${\bar{R}}_{1}\left(\Delta_{X}\right)=\underset{\left(R,\Delta_{X}\right)\in R_{1}}{\inf}R.$$

Wyner [2] characterized the infimum of all achievable rates ${\bar{R}}_{1}\left(\Delta_{X}\right)$ by the single-letter RDF $R_{X|Y}\left(\Delta_{X}\right)$ given by (1) and (3). The coding Theorems are given by Theorem 1 with $R^{*}\left(\Delta_{X}\right)$ and $\bar{R}\left(\Delta_{X}\right)$ replaced by ${\bar{R}}_{1}\left(\Delta_{X}\right)$ and $R_{X|Y}\left(\Delta_{X}\right)$, respectively. That is, ${\bar{R}}_{1}\left(\Delta_{X}\right)=R_{X|Y}\left(\Delta_{X}\right)$ (using Wyner’s notation [[2], Appendix A.1]) These coding theorems generalized earlier work of Berger [3] for finite alphabet spaces. Wyner also derived a fundamental lower bound on $R^{*}\left(\Delta_{X}\right)$ in terms of ${\bar{R}}_{1}\left(\Delta_{X}\right)$, as stated in the next remark.

**Remark 1.**  *Wyner [[2], Remarks, p. 65]*

*(A) For $Z\in M(\Delta_{X})$, $\hat{X}=f\left(Y,Z\right)$, and thus $P_{Z|X,Y}=P_{Z|X}$. Then, by a property of conditional mutual information and the data processing inequality:*

(36) $$\begin{array}{c} I\left(X;Z|Y\right)=I\left(X;Z,f\left(Y,Z\right)|Y\right)\geq I\left(X;\hat{X}|Y\right)\geq R_{X|Y}\left(\Delta_{X}\right), \end{array}$$

*where the last equality is defined since $\hat{X}\in M_{0}\left(\Delta_{X}\right)$ (see [[2], Remarks, p. 65]. Moreover, minimizing (36) over $Z\in M(\Delta_{X})$ gives*

(37) $$\begin{array}{c} R^{*}\left(\Delta_{X}\right)\geq R_{X|Y}\left(\Delta_{X}\right). \end{array}$$

*(B) Inequality (37) holds with equality, i.e., $R^{*}\left(\Delta_{X}\right)=R_{X|Y}\left(\Delta_{X}\right)$ if $\hat{X}\in M_{0}\left(\Delta_{X}\right)$, which achieves $I\left(X;\hat{X}|Y\right)=R_{X|Y}\left(\Delta_{X}\right)$ can be generated as in Figure 2 with $I\left(X;Z|Y\right)=I\left(X;\hat{X}|Y\right)$. This occurs if and only if $I(X;Z|\hat{X},Y)=0$, and follows from the identity and lower bound*

(38) $$\begin{array}{cc} I(X;Z|Y)= & I\left(X;Z,\hat{X}|Y\right)=I\left(X;Z|Y,\hat{X}\right)+I\left(X;\hat{X}|Y\right) \end{array}$$

(39) $$\begin{array}{cc} \geq & I(X;\hat{X}|Y), \end{array}$$

*where the inequality holds with equality if and only if $I(X;Z|\hat{X},Y)=0$.*

## 3. Main Theorems and Discussion

In this section, we state the main results of this paper. These are the achievable lower bounds of Lemma 1 and Theorem 2, which hold for RVs defined on general abstract alphabet spaces, and Theorems 4 and 5, which hold for multivariate Gaussian RVs.

### 3.1. Side Information at Encoder and Decoder for an Arbitrary Source

We start with the following achievable lower bound on the conditional mutual information $I(X;\hat{X}|Y)$, which appears in the definition of $R_{X|Y}\left(\Delta_{X}\right)$ of (1); this strengthens Gray’s lower bound (8) [[4], Theorem 3.1].

**Lemma 1.**  *Achievable lower bound on conditional mutual information. Let $\left(X,Y,\hat{X}\right)$ be a triple of arbitrary RVs taking values in the abstract spaces $X\times Y\times \hat{X}$, with distribution $P_{X,Y,\hat{X}}$ and joint marginal the fixed distribution $P_{X,Y}$ of (X,Y). Then, the following hold.*

*(a) The inequality holds:*

(40) $$\begin{array}{c} I\left(X;\hat{X}|Y\right)\geq I\left(X;\hat{X}\right)-I\left(X;Y\right). \end{array}$$

*Moreover, the equality holds*

(41) $$\begin{array}{c} I\left(X;\hat{X}|Y\right)=I\left(X;\hat{X}\right)-I\left(X;Y\right)\in \left[0,\infty \right), \end{array}$$

*if and only if*

(42) $$\begin{array}{c} P_{X|\hat{X},Y}=P_{X|\hat{X}}-a.s.\;or\;equivalently\;Y↔\hat{X}↔X\;is\;a\;MC. \end{array}$$

*(b) If $Y↔\hat{X}↔X$ is a Markov chain then the equality holds*

(43) $$\begin{array}{c} R_{X|Y}\left(\Delta_{X}\right)=R_{X}\left(\Delta_{X}\right)-I\left(X;Y\right),\;\Delta_{X}\in D_{C}\left(X|Y\right), \end{array}$$

*i.e., for all $\Delta_{X}$ that belong to strictly positive set $D_{C}\left(X|Y\right)\subseteq \left[0,\infty \right)$.*

**Proof.**  See Appendix A.1. □

The next theorem which holds for arbitrary RVs is further used to derive the characterization of $R_{X|Y}\left(\Delta_{X}\right)$ for multivariate Gaussian RVs.

**Theorem 2.**  *Achievable lower bound on conditional mutual information and mean-square error estimation*

*(a) Let $\left(X,Y,\hat{X}\right)$ be a triple of arbitrary RVs on the abstract spaces $X\times Y\times \hat{X}$, with distribution $P_{X,Y,\hat{X}}$ and joint marginal the fixed distribution $P_{X,Y}$ of (X,Y).*

*Define the conditional mean of X conditioned on $\left(\hat{X},Y\right)$ by*

(44) $$\begin{array}{c} {\bar{X}}^{cm}\overset{▵}{=}E\left(X|Y,\hat{X}\right)=e\left(Y,\hat{X}\right), \end{array}$$

*for some measurable function $f:Y\times \hat{X}\to X$.*

*(1) The inequality holds:*

(45) $$I\left(X;\hat{X}|Y\right)\geq I\left(X;{\bar{X}}^{cm}|Y\right).$$

*(2) The equality holds, $I\left(X;\hat{X}|Y\right)=I\left(X;{\bar{X}}^{cm}|Y\right)$ if anyone of the conditions (i) or (ii) holds.*

(46) $$\begin{array}{cc} \; & \left(i\right)\;{\bar{X}}^{cm}=\hat{X}-a.s\\ \; & \left(ii\right)\;For\;a\;fixed\;y\in Y\;the\;function\;e\left(y,\cdot \right):\hat{X}\to X,e\left(y,\hat{x}\right)={\bar{x}}^{cm}\;uniquely\;defines\;\hat{x} \end{array}$$

(47) $$\begin{array}{cc} \; & i.e.,e\left(y,\cdot \right)is\;an\;injective\;function\;on\;the\;support\;of\;\hat{x}. \end{array}$$

*(b) In part (a) let $\left(X,Y,\hat{X}\right)$ be a triple of arbitrary RVs on $X\times Y\times \hat{X}=R^{n_{x}}\times R^{n_{y}}\times R^{n_{x}}$, $\left(n_{x},n_{y}\right)\in Z_{+}\times Z_{+}$.*

*For all measurable functions $\left(y,\hat{x}\right)⟼g\left(y,\hat{x}\right)\in R^{n_{x}}$, the mean-square error satisfies*

(48) $$\begin{array}{c} E\left\{||X-g\left(Y,\hat{X}\right){\left|\right|}_{R^{n_{x}}}^{2}\right\}\geq E\left\{||X-E\left(X|Y,\hat{X}\right){\left|\right|}_{R^{n_{x}}}^{2}\right\},\;\forall g\left(\cdot \right). \end{array}$$

**Proof.**  See Appendix A.2. □

### 3.2. Side Information at Encoder and Decoder for Multivariate Gaussian Source

**Figure 3:**
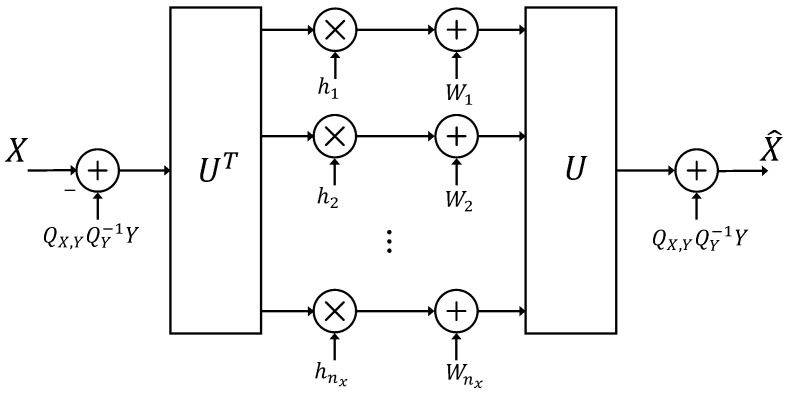
$R_{X|Y}\left(\Delta_{X}\right)$: A realization of optimal reproduction $\hat{X}$ over parallel additive Gaussian noise channels of Theorem 4, where $h_{i}\overset{▵}{=}1-\frac{\delta_{i}}{\lambda_{i}}\geq 0,i=1,\ldots ,n_{x}$ are the diagonal element of the spectral decomposition of the matrix $H=U\mathrm{diag}\left\{h_{1},\ldots ,h_{nx}\right\}U^{T}$, and $W_{i}\in N\left(0,h_{i}\delta_{i}\right),i=1,\ldots ,n_{x}$, the additive noise introduced due to compression.

The characterizations of the RDFs $R_{X|Y}\left(\Delta_{X}\right)$ and $\bar{R}\left(\Delta_{X}\right)$ for a multivariate Gaussian source are encapsulated in Theorems 3–5; these are proved in Section 4. These theorems include the structural properties of optimal test channels or realizations of $\left(\hat{X},Z\right)$, which induce joint distributions. Furthermore, they achieve the RDFs; the closed form expressions of the RDFs are based on a water-filling. The realization of the optimal test channel of $R_{X|Y}\left(\Delta_{X}\right)$ is shown in Figure 3.

The following theorem gives a parametric realization of optimal test channel that achieves the characterization of the RDF $R_{X|Y}\left(\Delta_{X}\right)$.

**Theorem 3.**  *Characterization of $R_{X|Y}\left(\Delta_{X}\right)$ by test channel realization. Consider the RDF $R_{X|Y}\left(\Delta_{X}\right)$ defined by (1), for the multivariate Gaussian source with mean-square error distortion defined by (9)–(18). The following hold.*

*(a) The optimal realization $\hat{X}$ that achieves $R_{X|Y}\left(\Delta_{X}\right)$ is parametrized by the matrices $\left(H,Q_{W}\right)$ and represented by*

(49) $$\begin{array}{cc} \hat{X} & =H\left(X-Q_{X,Y}Q_{Y}^{-1}Y\right)+Q_{X,Y}Q_{Y}^{-1}Y+W \end{array}$$

(50) $$\begin{array}{cc} \; & =H\left(X-Q_{X,Y}Q_{Y}^{-1}Y\right)+Q_{X,Y}Q_{Y}^{-1}Y+H\Psi ,\;ifH^{-1}\;exists, \end{array}$$

*where*

(51) $$\begin{array}{cc} \; & HQ_{X|Y}=Q_{X|Y}H^{T}\overset{▵}{=}Q_{X|Y}-\Sigma_{\Delta }⪰0, \end{array}$$

(52) $$\begin{array}{cc} \; & W\;idependent\;of\;(X,Y),\;Q_{W}\in N\left(0,Q_{W}\right), \end{array}$$

(53) $$\begin{array}{cc} \; & Q_{W}\overset{▵}{=}HQ_{X|Y}-HQ_{X|Y}H^{T}=H\Sigma_{\Delta }=\Sigma_{\Delta }-\Sigma_{\Delta }Q_{X|Y}^{-1}\Sigma_{\Delta }=\Sigma_{\Delta }H⪰0, \end{array}$$

(54) $$\begin{array}{cc} \; & W=H\Psi ,\;\Psi \in N\left(0,Q_{\Psi }\right),\;Q_{\Psi }\overset{▵}{=}\Sigma_{\Delta }H^{-1}=H^{-1}\Sigma_{\Delta },\;if\;H^{-1}\;exists, \end{array}$$

(55) $$\begin{array}{cc} \; & \Sigma_{\Delta }\overset{▵}{=}E\left\{\left(X-\hat{X}\right){\left(X-\hat{X}\right)}^{T}\right\}, \end{array}$$

(56) $$\begin{array}{cc} \; & Q_{\hat{X}|Y}=Q_{X|Y}-\Sigma_{\Delta }⪰0, \end{array}$$

(57) $$\begin{array}{cc} \; & Q_{X|Y}=Q_{X}-Q_{X,Y}Q_{Y}^{-1}Q_{X,Y}^{T}≻0,\;Q_{X,Y}=Q_{X}C^{T},\;\;Q_{Y}=CQ_{X}C^{T}+DD^{T}. \end{array}$$

*Moreover, the optimal parametric realization of $\hat{X}$ satisfies the following structural properties.*

(58) $$\begin{array}{cc} \; & \left(i\right)\;P_{X|\hat{X},Y}=P_{X|\hat{X}},\;if\;Q_{X}≻\Sigma_{\Delta }, \end{array}$$

(59) $$\begin{array}{cc} \; & \left(ii\right)\;E\left\{X|Y\right\}=E\left\{\hat{X}|Y\right\},\;if\;Q_{X}⪰\Sigma_{\Delta }, \end{array}$$

(60) $$\begin{array}{cc} \; & \left(iii\right)\;\mathrm{cov}\left(X,\hat{X}|Y\right)=\mathrm{cov}\left(\hat{X},\hat{X}|Y\right),\;if\;Q_{X|Y}≻\Sigma_{\Delta }, \end{array}$$

(61) $$\begin{array}{cc} \; & \left(iv\right)\;E\left\{X|\hat{X},Y\right\}=E\left\{X|\hat{X}\right\}=\hat{X},\;if\;Q_{X|Y}≻\Sigma_{\Delta }. \end{array}$$

*(b) The RDF $R_{X|Y}\left(\Delta_{X}\right)$ is given by*

(62) $$R_{X|Y}\left(\Delta_{X}\right)=\underset{\Sigma_{\Delta }⪰0,\;Q_{X|Y}-\Sigma_{\Delta }⪰0,\;\mathrm{trace}\left(\Sigma_{\Delta }\right)\leq \Delta_{X}}{\inf}\frac{1}{2}\log\max\left\{1,\det(Q_{X|Y}\Sigma_{\Delta }^{-1})\right\}.$$

**Proof.**  The proof is given in Section 4. □

The next theorem gives additional structural properties of the optimal test channel realization of Theorem 3 and uses these properties to characterize RDF $R_{X|Y}\left(\Delta_{X}\right)$ via a water-filling solution.

**Theorem 4.**  *Characterization of $R_{X|Y}\left(\Delta_{X}\right)$ via water-filling solution. Consider the RDF $R_{X|Y}\left(\Delta_{X}\right)$ defined by (1), for the multivariate Gaussian source with mean-square error distortion defined by (9)–(18), and its characterization in Theorem 3. The following hold.*

*(a) The matrices of the parametric realization of $\hat{X}$,*

(63) $$\begin{array}{cc} \{\Sigma_{\Delta }, & Q_{X|Y},H,Q_{W}\}\;have\;spectral\\ \; & decompositions\;with\;respect\;to\;the\;same\;unitary\;matrix\;UU^{T}=I_{n_{x}},U^{T}U\;=I_{n_{x}}, \end{array}$$

*where the realization coefficients are*

(64) $$\begin{array}{cc} \; & Q_{W}=H\Sigma_{\Delta }=U\mathrm{diag}\left(\sigma_{W_{1}}^{2},\cdots ,\sigma_{W_{n_{x}}}^{2}\right)U^{T},\;\Sigma_{\Delta }=U\mathrm{diag}\left(\delta_{1},\cdots ,\delta_{n_{x}}\right)U^{T}, \end{array}$$

(65) $$\begin{array}{cc} \; & H=I_{n_{x}}-Q_{X|Y}^{-1}\Sigma_{\Delta }=U\mathrm{diag}\left(h_{1},\cdots ,h_{n_{x}}\right)U^{T},\;Q_{X|Y}=U\mathrm{diag}\left(\lambda_{1},\cdots ,\lambda_{n_{x}}\right)U^{T}, \end{array}$$

(66) $$\begin{array}{cc} \; & \lambda_{1}\geq \lambda_{2}\geq \cdots \geq \lambda_{n_{x}}>0,\;\delta_{1}\geq \delta_{2}\geq \ldots \geq \delta_{n_{x}}>0, \end{array}$$

(67) $$\begin{array}{cc} \; & \sigma_{W_{1}}^{2}\geq \sigma_{W_{2}}^{2}\geq \ldots \geq \sigma_{W_{n_{x}}}\geq 0,\;h_{1}\geq h_{2}\geq \cdots \geq h_{n_{x}}\geq 0,\;\sigma_{W_{i}}^{2}=h_{i}\delta_{i},\;h_{i}\overset{▵}{=}1-\frac{\delta_{i}}{\lambda_{i}}, \end{array}$$

*and the eigenvalues $\sigma_{W_{i}}^{2}$ and $h_{i}$ are given by*

(68) $$\begin{array}{cc} \; & \sigma_{W_{i}}^{2}=\frac{\min\left(\lambda_{i},\delta_{i}\right)\left(\lambda_{i}-\min\left(\lambda_{i},\delta_{i}\right)\right)}{\lambda_{i}},\;h_{i}=\frac{\lambda_{i}-\min\left(\lambda_{i},\delta_{i}\right)}{\lambda_{i}},\;\sum_{i=1}^{n_{x}}\min\left(\lambda_{i},\delta_{i}\right)=\Delta_{X}. \end{array}$$

*Moreover, if $\sigma_{W_{i}}^{2}=0$, then $h_{i}=0$, and vice versa.*

*(b) The RDF $R_{X|Y}\left(\Delta_{X}\right)$ is given by the water-filling solution:*

(69) $$R_{X|Y}\left(\Delta_{X}\right)=\frac{1}{2}\log\max\left\{1,\det(Q_{X|Y}\Sigma_{\Delta }^{-1})\right\}=\frac{1}{2}\sum_{i=1}^{n_{x}}\log\frac{\lambda_{i}}{\delta_{i}},$$

*where*

(70) $$E\left\{||X-\hat{X}{\left|\right|}_{R^{n_{x}}}^{2}\right\}=\mathrm{trace}\left(\Sigma_{\Delta }\right)=\sum_{i=1}^{n_{x}}\delta_{i}=\Delta_{X},\;\delta_{i}=\left\{\begin{array}{ccc} \mu , & if & \mu <\lambda_{i}\\ \lambda_{i}, & if & \mu \geq \lambda_{i} \end{array}\right.$$

*and μ∈(0,∞) is a Lagrange multiplier (obtained from the Kuch–Tucker conditions).*

*(c) Figure 3 depicts the parallel channel scheme that realizes the optimal $\hat{X}$ of parts (a), (b), which achieves $R_{X|Y}\left(\Delta_{X}\right)$.*

*(d) If X and Y are independent or Y is replaced by a RV that generates the trivial information, i.e., the σ−algebra of Y is σ{Y}={Ω,∅} (or C=0 in (15)), then (a)–(c) hold with $Q_{X|Y}=Q_{X},Q_{X,Y}=0$, and $R_{X|Y}\left(\Delta_{X}\right)=R_{X}\left(\Delta_{X}\right)$, i.e., reduces to the marginal RDF of X.*

**Proof.**  The proof is given in Section 4. □

The proof of Theorem 4 (see Section 4) is based on the identification of structural properties of the test channel distribution. Some of the implications are briefly described below.

*Conclusion 1:* The construction and the structural properties of the optimal test channel $P_{X|\hat{X},Y}$ that achieves the water-filling characterization of the RDF $R_{X|Y}\left(\Delta_{X}\right)$ of Theorems 3 and 4 are not documented elsewhere in the literature.

(i) Structural properties (58) and (61) strengthen Gray’s inequality [[4], Theorem 3.1], (see proof of (8)) to the equality. That is, structural property (58) implies that Gray’s [[4], Theorem 3.1] lower bound (8) holds with equality for a strictly positive surface (See Gray [4] for definition) $\Delta_{X}\in D_{C}\left(X|Y\right)\subseteq \left[0,\infty \right)$, i.e.,

(71) $$\begin{array}{c} R_{X|Y}\left(\Delta_{X}\right)=R_{X}\left(\Delta_{X}\right)-I\left(X;Y\right),\;\Delta_{X}\in D_{C}\left(X|Y\right)\overset{▵}{=}\left\{\Delta_{X}\in \left[0,\infty \right):\Delta_{X}\leq n_{x}\lambda_{n_{x}}\right\}. \end{array}$$

The set $D_{C}\left(X|Y\right)$ excludes values of $\Delta_{X}\in \left[0,\infty \right)$ for which water-filling is active in (69) and (70).

By the realization of the optimal reproduction $\hat{X}$, it follows that the subtraction of equal quantities $E\left\{X|Y\right\}$ at the encoder and decoder does not affect the information measure, noting that $E\left\{X|Y\right\}=E\left\{\hat{X}|Y\right\}$.

Theorem 4 points (a) and (b) are obtained with the aid of Theorem 3 and Hadamard’s inequality, which shows $Q_{X|Y}$ and $\Sigma_{\Delta }$ have the same eigenvectors.

(ii) Structural properties of realizations of Theorems 3 and 4: The matrices $\left\{\Sigma_{\Delta },Q_{X|Y},H,Q_{W}\right\}$ are nonnegative symmetric and have a spectral decomposition with respect to the same unitary matrix $UU^{T}=I_{n_{x}}$ [21]. This implies that the test channel is equivalently represented by parallel additive Gaussian noise channels (subject to pre-processing and post-processing at the encoder and decoder).

(iii) In Remark 4, we show that the realization of optimal $\hat{X}$ in Figure 3, which achieves the RDF of Theorem 4, degenerates to Wyner’s [2] optimal realization, which attains the RDF $R_{X|Y}\left(\Delta_{X}\right)$, for the tuple of scalar-valued, jointly Gaussian RVs (X,Y) with square error distortion function.

### 3.3. Side Information Only at Decoder for Multivariate Gaussian Source

Theorem 5 gives the optimal test channel that achieves the characterization of the RDF $\bar{R}\left(\Delta_{X}\right)$ and further states that there is no loss of compression rate if side information is only available at the decoder. That is, although in general, $\bar{R}\left(\Delta_{X}\right)\geq R_{X|Y}\left(\Delta_{X}\right)$, an optimal reproduction $\hat{X}=f\left(Y,Z\right)$ of *X*, where f(·,·) is linear, is constructed such that the inequality holds with equality.

**Theorem 5.**  *Characterization and water-filling solution of $\bar{R}\left(\Delta_{X}\right)$. Consider the RDF $\bar{R}\left(\Delta_{X}\right)$ defined by (5) for the multivariate Gaussian source with mean-square error distortion, defined by (9)–(18). Then, the following hold.*

*(a) The characterization of the RDF, $\bar{R}\left(\Delta_{X}\right)$ satisfies*

(72) $$\begin{array}{c} \bar{R}\left(\Delta_{X}\right)\geq R_{X|Y}\left(\Delta_{X}\right), \end{array}$$

*where $R_{X|Y}\left(\Delta_{X}\right)$ is given in Theorem 4b.*

*(b) The optimal realization $\hat{X}=f\left(Y,Z\right)$, which achieves the lower bound in (72), i.e., $\bar{R}\left(\Delta_{X}\right)=R_{X|Y}\left(\Delta_{X}\right)$, is represented by*

(73) $$\begin{array}{cc} \hat{X}= & f(Y,Z) \end{array}$$

(74) $$\begin{array}{cc} = & \left(I-H\right)Q_{X,Y}Q_{Y}^{-1}Y+Z, \end{array}$$

(75) $$\begin{array}{cc} Z= & HX+W, \end{array}$$

(76) $$\begin{array}{cc} (H, & Q_{W})\;given\;by\;(51)–(57),\;and\;(63)\;holds. \end{array}$$

*Moreover, the following structural properties hold:*

*(1) The optimal test channel satisfies*

(77) $$\begin{array}{cc} \; & \left(i\right)\;P_{X|\hat{X},Y,Z}=P_{X|\hat{X},Y}\overset{\left(\alpha \right)}{=}P_{X|\hat{X}},\;where\left(\alpha \right)holds\;if\;Q_{X}≻\Sigma_{\Delta }, \end{array}$$

(78) $$\begin{array}{cc} \; & \left(ii\right)\;E\left\{X|\hat{X},Y,Z\right\}=E\left\{X|\hat{X},Y\right\}\overset{\left(\beta \right)}{=}E\left\{X|\hat{X}\right\}\overset{\left(\gamma \right)}{=}\hat{X},\;where\left(\beta \right),\left(\gamma \right)hold\;if\;Q_{X|Y}≻\Sigma_{\Delta }, \end{array}$$

(79) $$\begin{array}{cc} \; & \left(iii\right)\;P_{Z|X,Y}=P_{Z|X}. \end{array}$$

*(2) Structural property (2) of Theorem 4a holds.*

**Proof.**  It is given in Section 4. □

The proof of Theorem 5 is based on the derivation of the structural properties and Theorem 4. Some implications are discussed below.

*Conclusion 2:* The optimal reproduction $\hat{X}=f\left(X,Z\right)$ or test channel distribution $P_{X|\hat{X},Y,Z}$, which achieves $\bar{R}\left(\Delta_{X}\right)$ of Theorem 5, are not reported in the literature.

(i) From the structural property (1) of Theorem 5, i.e., (77), it follows that the lower bound $\bar{R}\left(\Delta_{X}\right)\geq R_{X|Y}\left(\Delta_{X}\right)$ is achieved by the realization $\hat{X}=f\left(Y,Z\right)$ of Theorem 5b; i.e., for a given Y=y, then $\hat{X}$ uniquely defines *Z*.

(ii) If *X* is independent of *Y* or *Y* generates trivial information, then the RDFs $\bar{R}\left(\Delta_{X}\right)={\bar{R}}_{X|Y}\left(\Delta_{X}\right)$ degenerate to the classical RDF of the source *X*, i.e., $R_{X}\left(\Delta_{X}\right)$, as expected. This is easily verified from (73) and (76), i.e., $Q_{X,Y}=0$, which implies $\hat{X}=Z$.

For scalar-valued RVs, $X:\Omega \to R,Y:\Omega \to R,X\in N(0,\sigma_{X}^{2})$, and *X* independent of *Y*, then the optimal realization reduces to

(80) $$\begin{array}{cc} \; & \hat{X}=Z=\left(1-\frac{\Delta_{X}}{\sigma_{X}^{2}}\right)X+\sqrt{\left(1-\frac{\Delta_{X}}{\sigma_{X}^{2}}\right)\Delta_{X}}\bar{W},\;\bar{W}\in N\left(0,1\right),\;\sigma_{X}^{2}\leq \Delta_{X}, \end{array}$$

(81) $$\begin{array}{cc} \; & Q_{\hat{X}}=Q_{Z}=\sigma_{\hat{X}}^{2}=\sigma_{X}^{2}-\Delta_{X}\geq 0, \end{array}$$

as expected.

(iii) In Remark 4, we show that the realization of optimal $\hat{X}=f\left(Y,Z\right)$, which achieves the RDF $\bar{R}\left(\Delta_{X}\right)$ of Theorem 5, degenerates to Wyner’s [2] realization that attains the RDF $\bar{R}\left(\Delta_{X}\right)$, of the tuple of scalar-valued, jointly Gaussian RVs (X,Y), with the square error distortion function.

## 4. Proofs of Theorems 3–5

In this section, we derive the statements of Theorems 3–5 by making use of Theorem 2 (which holds for general abstract alphabet spaces) by restricting attention to multivariate jointly Gaussian (X,Y).

### 4.1. Side Information at Encoder and Decoder

For jointly Gaussian RVs $\left(X,Y,\hat{X}\right)$, in the next theorem we identify simple sufficient conditions for the lower bound of Theorem 2 to be achievable.

**Theorem 6.**  *Sufficient conditions for the lower bounds of Theorem 2 to be achievable. Consider the statement of Theorem 2 for a triple of jointly Gaussian RVs $\left(X,Y,\hat{X}\right)$ on $R^{n_{x}}\times R^{n_{y}}\times R^{n_{x}}$, $\left(n_{x},n_{y}\right)\in Z_{+}\times Z_{+}$, i.e., $P_{X,Y,\hat{X}}=P_{X,Y,\hat{X}}^{G}$ and joint marginal the fixed Gaussian distribution $P_{X,Y}=P_{X,Y}^{G}$ of (X,Y)*

*Then,*

(82) $$\begin{array}{cc} \; & {\bar{X}}^{cm}\overset{▵}{=}E\left(X|Y,\hat{X}\right)=e^{G}\left(Y,\hat{X}\right), \end{array}$$

(83) $$\begin{array}{cc} \; & e^{G}\left(Y,\hat{X}\right)=E\left(X|Y\right)+\mathrm{cov}\left(X,\hat{X}|Y\right){\left\{\mathrm{cov}(\hat{X},\hat{X}|Y)\right\}}^{†}\left(\hat{X}-E\left(\hat{X}|Y\right)\right). \end{array}$$

*Moreover, the following hold.*

*Case (i). $\mathrm{cov}(\hat{X},\hat{X}|Y)≻0$, that is, $\mathrm{rank}\left(Q_{\hat{X}|Y}\right)=n_{x}$. Condition (84) is sufficient for $I\left(X;\hat{X}|Y\right)=I\left(X;{\bar{X}}^{cm}|Y\right)$.*

(84) $$\begin{array}{c} {\bar{X}}^{cm}\overset{▵}{=}E\left(X|Y,\hat{X}\right)=e^{G}\left(Y,\hat{X}\right)=\hat{X}-a.s. \end{array}$$

*In addition, Conditions 1 and 2 below are sufficient for (84) to hold.*

(85) $$\begin{array}{cc} \; & Condition\;1.\;E\left(X|Y\right)=E\left(\hat{X}|Y\right) \end{array}$$

(86) $$\begin{array}{cc} \; & Condition\;2.\;\mathrm{cov}\left(X,\hat{X}|Y\right)\mathrm{cov}{\left(\hat{X},\hat{X}|Y\right)}^{-1}=I_{n_{x}} \end{array}$$

*Case (ii). $\mathrm{cov}(\hat{X},\hat{X}|Y)⪰0$ but not $\mathrm{cov}(\hat{X},\hat{X}|Y)≻0$; that is, $\mathrm{rank}\left(Q_{\hat{X}|Y}\right)=n_{1}<n_{x}$. Condition (87) is sufficient for $I\left(X;\hat{X}|Y\right)=I\left(X;{\bar{X}}^{cm}|Y\right)$.*

(87) $$\begin{array}{c} e^{G}\left(\cdot ,\cdot \right)\;defined\;by\;(83)\;satisfies\;(47). \end{array}$$

*In addition, a sufficient condition for (87) to hold is, for a fixed Y=y∈Y, the σ−algebras satisfy $F^{\hat{X}}=F^{e^{G}\left(y,\hat{X}\right)}$.*

**Proof.**  Note that identity (83) follows from Proposition 1, (26), by letting $Y=\hat{X}$ and G be the information generated by *Y*. Consider Case (i); If (84) holds then $I(X;\hat{X}|{\bar{X}}^{cm},Y)=0$. By (83), Conditions 1 and 2 are sufficient for (84) to hold. Consider Case (ii). Sufficient condition (87) follows from Theorem 2, and implies $I(X;\hat{X}|{\bar{X}}^{cm},Y)=0$. The statement below (87) follows from Proposition 2. □

Now, we turn our attention to the optimization problem $R_{X|Y}\left(\Delta_{X}\right)$ defined by (1) for the multivariate Gaussian source with mean-square error distortion defined by (9)–(18). In the next lemma, we derive a *preliminary parametrization* of the optimal reproduction distribution $P_{\hat{X}|X,Y}$ of the RDF $R_{X|Y}\left(\Delta_{X}\right)$.

**Lemma 2.**  *Preliminary parametrization of optimal reproduction distribution of $R_{X|Y}\left(\Delta_{X}\right)$. Consider the RDF $R_{X|Y}\left(\Delta_{X}\right)$ defined by (1) for the multivariate Gaussian source, i.e., $P_{X,Y}=P_{X,Y}^{G}$, with mean-square error distortion defined by (9)–(18).*

*(a) For every joint distribution $P_{X,Y,\hat{X}}$ there exists a jointly Gaussian distribution denoted by $P_{X,Y,\hat{X}}^{G}$, with marginal the fixed distribution $P_{X,Y}^{G}$, which minimizes $I(X;\hat{X}|Y)$ and satisfies the average distortion constraint, i.e., with $d_{X}\left(x,\hat{x}\right)=||x-\hat{x}{\left|\right|}_{R^{n_{x}}}^{2}$.*

*(b) The conditional reproduction distribution of the RDF $R_{X|Y}\left(\Delta_{X}\right)$ is $P_{\hat{X}|X,Y}=P_{\hat{X}|X,Y}^{G}$ and induced by the parametric realization of $\hat{X}$ (in terms of $H,G,Q_{W}$),*

(88) $$\begin{array}{cc} \; & \hat{X}=HX+GY+W, \end{array}$$

(89) $$\begin{array}{cc} \; & H\in R^{n_{x}\times n_{x}},\;G\in R^{n_{x}\times n_{y}}, \end{array}$$

(90) $$\begin{array}{cc} \; & W\in N\left(0,Q_{W}\right),\;Q_{W}⪰0, \end{array}$$

(91) $$\begin{array}{cc} \; & W\;independent\;of\;(X,Y), \end{array}$$

*and $\hat{X}$ is a Gaussian RV.*

*(c) $R_{X|Y}\left(\Delta_{X}\right)$ is characterized by the optimization problem.*

(92) $$\begin{array}{cc} R_{X|Y}\left(\Delta_{X}\right)\overset{▵}{=} & \underset{M_{0}^{G}\left(\Delta_{X}\right)}{\inf}I\left(X;\hat{X}|Y\right),\;\Delta_{X}\in \left[0,\infty \right), \end{array}$$

*where $M_{0}^{G}\left(\Delta_{X}\right)$ is specified by the set*

(93) $$\begin{array}{cc} M_{0}^{G}\left(\Delta_{X}\right)\overset{▵}{=} & \left\{\hat{X}:\Omega \to \hat{X}:\left(88)-(91\right)\;hold,\;and\;E\left\{||X-\hat{X}{\left|\right|}_{R^{n_{x}}}^{2}\right\}\leq \Delta_{X}\right\}. \end{array}$$

*(d) If there exists $\left(H,G,Q_{W}\right)$ such that (84) or (87) hold, then a further lower bound on $R_{X|Y}\left(\Delta_{X}\right)$ is achieved in the subset $M_{0}^{G,o}\left(\Delta_{X}\right)\subseteq M_{0}^{G}\left(\Delta_{X}\right)$ defined by*

(94) $$\begin{array}{cc} M_{0}^{G,o}\left(\Delta_{X}\right)\overset{▵}{=} & \{\hat{X}:\Omega \to \hat{X}:\;\left(88\right)–\left(91\right)\;hold,\;(84)\;or\;(87)\;hold,\\ \; & E\left\{||X-\hat{X}{\left|\right|}_{R^{n_{x}}}^{2}\right\}\leq \Delta_{X}\}, \end{array}$$

*and the corresponding characterization of the RDF is*

(95) $$\begin{array}{cc} R_{X|Y}\left(\Delta_{X}\right)\overset{▵}{=} & \underset{M_{0}^{G,o}\left(\Delta_{X}\right)}{\inf}I\left(X;\hat{X}|Y\right),\;\Delta_{X}\in \left[0,\infty \right). \end{array}$$

**Proof.**  (a) This is omitted since it is similar to the classical unconditional RDF $R_{X}\left(\Delta_{X}\right)$ of a Gaussian message $X\in N(0,Q_{X})$. (b) By (a), the conditional distribution $P_{\hat{X}|X,Y}^{G}$ is such that, its conditional mean is linear in (X,Y), its conditional covariance is nonrandom, i.e., constant, and for fixed (X,Y)=(x,y), $P_{\hat{X}|X,Y}^{G}$ is Gaussian. Such a distribution is induced by the parametric realization (88)–(91). (c) Follows from parts (a) and (b). (d) Follows from Theorem 6 and (48) due to the achievability of the lower bounds. □

In the next theorem, we identify the optimal triple $\left(H,G,Q_{W}\right)$ such that (84) or (87) hold (i.e., establish its existence), characterize the RDF by $R_{X|Y}\left(\Delta_{X}\right)\overset{▵}{=}\inf_{M_{0}^{G,o}\left(\Delta_{X}\right)}I\left(X;\hat{X}|Y\right)$, and construct a realization $\hat{X}$ that achieves it.

**Theorem 7.**  *Characterization of RDF $R_{X|Y}\left(\Delta_{X}\right)$. Consider the RDF $R_{X|Y}\left(\Delta_{X}\right)$, defined by (1), for the multivariate Gaussian source with mean-square error distortion, defined by (9)–(18). The characterization of the RDF $R_{X|Y}\left(\Delta_{X}\right)$ is*

(96) $$\begin{array}{cc} R_{X|Y}\left(\Delta_{X}\right)\overset{▵}{=} & \underset{Q(\Delta_{X})}{\inf}I\left(X;\hat{X}|Y\right) \end{array}$$

(97) $$\begin{array}{cc} = & \underset{M_{0}^{G,o}\left(\Delta_{X}\right)}{\inf}I\left(X;\hat{X}|Y\right) \end{array}$$

(98) $$\begin{array}{cc} = & \underset{Q(\Delta_{X})}{\inf}\frac{1}{2}\log\left\{\det(Q_{X|Y}\Sigma_{\Delta }^{-1})\right\}, \end{array}$$

*where*

(99) $$\begin{array}{cc} Q\left(\Delta_{X}\right)\overset{▵}{=} & \left\{\Sigma_{\Delta }⪰0:\;Q_{X|Y}-\Sigma_{\Delta }⪰0,\;\mathrm{trace}\left(\Sigma_{\Delta }\right)\leq \Delta_{X}\right\}, \end{array}$$

(100) $$\begin{array}{cc} \Sigma_{\Delta }\overset{▵}{=} & E\left\{\left(X-\hat{X}\right){\left(X-\hat{X}\right)}^{T}\right\}, \end{array}$$

(101) $$\begin{array}{cc} Q_{X|Y}= & Q_{X}-Q_{X,Y}Q_{Y}^{-1}Q_{X,Y}^{T}, \end{array}$$

(102) $$\begin{array}{cc} Q_{X,Y}= & Q_{X}C^{T},\;Q_{Y}=CQ_{X}C^{T}+DD^{T}. \end{array}$$

*The realization of the optimal reproduction $\hat{X}\in M_{0}^{G,o}\left(\Delta_{X}\right)$, which achieves $R_{X|Y}\left(\Delta_{X}\right)$, is given in Theorem 3a, also satisfies the properties stated under Theorem 3a. (i)–(iv).*

**Proof.**  See Appendix A.3. □

**Remark 2.**  *Structural properties of the optimal realization of Theorem 4a. For the characterization of the RDF $R_{X|Y}\left(\Delta_{X}\right)$ of Theorem 7, which is achieved by $\hat{X}$ defined in Theorem 3a in terms of the matrices $\left\{\Sigma_{\Delta },Q_{X|Y},H,Q_{W}\right\}$, we show in Corollary 2, the statements of Theorem 4a, i.e.,*

(103) $$\begin{array}{cc} \left(i\right) & \;H=H^{T}⪰0, \end{array}$$

(104) $$\begin{array}{cc} \left(ii\right) & \;\left\{\Sigma_{\Delta },\Sigma_{X|Y},H,Q_{W}\right\}\;have\;spectral\;repres.\;with\;respect\;to\;the\;same\;unitary\;matrix\;UU^{T}=I_{n_{x}}. \end{array}$$

To prove the structural property of Remark 2, we use the next corollary, which is a degenerate case of [[22], Lemma 2] (i.e., the structural properties of test channel of Gorbunov and Pinsker [23] nonanticipatory RDF of Markov sources).

**Corollary 1.**  *Structural properties of realization of optimal $\hat{X}$ of Theorem 4a. Consider the characterization of the RDF $R_{X|Y}\left(\Delta_{X}\right)$ of Theorem 7. Suppose $Q_{X|Y}≻0$ and $\Sigma_{\Delta }⪰0$ commute, that is,*

(105) $$\begin{array}{c} Q_{X|Y}\Sigma_{\Delta }=\Sigma_{\Delta }Q_{X|Y}. \end{array}$$

*Then,*

(106) $$\begin{array}{cc} \left(1\right) & \;H=I_{n_{x}}-\Sigma_{\Delta }Q_{X|Y}^{-1}=H^{T},\;Q_{W}=\Sigma_{\Delta }H^{T}=\Sigma_{\Delta }H=H\Sigma_{\Delta }=Q_{W}^{T}⪰0\\ \left(2\right) & \;\left\{\Sigma_{\Delta },Q_{X|Y},H,Q_{W}\right\}\;have\;spectral \end{array}$$

(107) $$\begin{array}{cc} \; & decompositions\;with\;respect\;to\;the\;same\;unitary\;matrix\;UU^{T}=I_{n_{x}},\;U^{T}U=I_{n_{x}}. \end{array}$$

*that is, the following hold.*

(108) $$\begin{array}{cc} \; & Q_{X|Y}=U\mathrm{diag}\left\{\lambda_{1},\cdots ,\lambda_{n_{x}}\right\}U^{T},\;\lambda_{1}\geq \lambda_{2}\geq \ldots \geq \lambda_{n_{x}}>0, \end{array}$$

(109) $$\begin{array}{cc} \; & \Sigma_{\Delta }=U\mathrm{diag}\left\{\delta_{1},\cdots ,\delta_{n_{x}}\right\}U^{T},\;\delta_{1}\geq \delta_{2}\geq \ldots \geq \delta_{n_{x}}\geq 0, \end{array}$$

(110) $$\begin{array}{cc} \; & H=U\mathrm{diag}\left\{1-\frac{\delta_{1}}{\lambda_{1}},\cdots ,1-\frac{\delta_{n_{x}}}{\lambda_{n_{x}}}\right\}U^{T}, \end{array}$$

(111) $$\begin{array}{cc} \; & Q_{W}=U\mathrm{diag}\left\{(1-\frac{\delta_{1}}{\lambda_{1}})\delta_{1},\cdots ,(1-\frac{\delta_{n_{x}}}{\lambda_{n_{x}}})\delta_{n_{x}}\right\}U^{T},\;\mathrm{and}\;(1-\frac{\delta_{k}}{\lambda_{k}})\delta_{k}\geq 0. \end{array}$$

**Proof.**  See Appendix A.4. □

In the next corollary, we re-express the realization of $\hat{X}$ of Theorem 4a, which characterizes the RDF of Theorem 7 using a translation of *X* and $\hat{X}$ by subtracting their conditional means with respect to *Y*, making use of property $E\left\{X|Y\right\}=E\left\{\hat{X}|Y\right\}$ of (78). This is the the realization shown in Figure 3.

**Corollary 2.**  *Equivalent characterization of $R_{X|Y}\left(\Delta_{X}\right)$. Consider the characterization of the RDF $R_{X|Y}\left(\Delta_{X}\right)$ of Theorem 7 and the realization of $\hat{X}$ of Theorem 3a and Theorem 4a. Define the translated RVs*

(112) $$X\overset{▵}{=}X-E\left\{X|Y\right\}=X-Q_{X,Y}Q_{Y}^{-1}Y,\;\hat{X}\overset{▵}{=}\hat{X}-E\left\{X|Y\right\}=\hat{X}-Q_{X,Y}Q_{Y}^{-1}Y.$$

*Let*

(113) $$\begin{array}{cc} \; & Q_{X|Y}=U\mathrm{diag}\left\{\lambda_{1},\cdots ,\lambda_{n_{x}}\right\}U^{T},\;UU^{T}=I_{n_{x}},U^{T}U=I_{n_{x}},\;\lambda_{1}\geq \lambda_{2}\geq \ldots \geq \lambda_{n_{x}}, \end{array}$$

(114) $$\begin{array}{cc} \; & \bar{X}\overset{▵}{=}U^{T}X,\;\hat{\bar{X}}\overset{▵}{=}U^{T}\hat{X}. \end{array}$$

*Then,*

(115) $$\begin{array}{cc} \; & \hat{X}=HX+W, \end{array}$$

(116) $$\begin{array}{cc} \; & I\left(X;\hat{X}|Y\right)=I\left(X;\hat{X}\right)=I\left(U^{T}X;U^{T}\hat{X}\right), \end{array}$$

(117) $$\begin{array}{cc} \; & E∥X-\hat{X}{∥}_{R^{n_{x}}}^{2}=E∥X-\hat{X}{∥}_{R^{n_{x}}}^{2}=E∥U^{T}X-U^{T}\hat{X}{∥}_{R^{n_{x}}}^{2}=\mathrm{trace}\left(\Sigma_{\Delta }\right), \end{array}$$

*where $\left(H,Q_{W}\right)$ are given in Theorem 3a.*

*Further, the characterization of the RDF $R_{X|Y}\left(\Delta_{X}\right)$ (98) satisfies the following equalities and inequality:*

(118) $$\begin{array}{cc} R_{X|Y}\left(\Delta_{X}\right)\overset{▵}{=} & \underset{Q(\Delta_{X})}{\inf}I\left(X;\hat{X}|Y\right)=\underset{Q(\Delta_{X})}{\inf}\frac{1}{2}\log\max\left\{1,\det(Q_{X|Y}\Sigma_{\Delta }^{-1})\right\} \end{array}$$

(119) $$\begin{array}{cc} = & \underset{E∥X-\hat{X}{∥}_{R^{n_{x}}}^{2}\leq \Delta_{X}}{\inf}I\left(X;\hat{X}\right) \end{array}$$

(120) $$\begin{array}{cc} = & \underset{E∥U^{T}X-U^{T}\hat{X}{∥}_{R^{n_{x}}}^{2}\leq \Delta_{X}}{\inf}I\left(U^{T}X;U^{T}\hat{X}\right) \end{array}$$

(121) $$\begin{array}{cc} \geq & \underset{E∥U^{T}X-U^{T}\hat{X}{∥}_{R^{n_{x}}}^{2}\leq \Delta_{X}}{\inf}\sum_{t=1}^{n_{x}}I\left({\bar{X}}_{t};{\hat{\bar{X}}}_{t}\right) \end{array}$$

*Moreover, the inequality (121) is achieved if $Q_{X|Y}⪰0$ and $\Sigma_{\Delta }⪰0$ commute; that is, if (105) holds, then*

(122) $$R_{X|Y}\left(\Delta_{X}\right)=\underset{\sum_{i=1}^{n_{x}}\delta_{i}\leq \Delta_{X}}{\inf}\frac{1}{2}\sum_{i=1}^{n_{x}}\log\max\left\{1,\frac{\lambda_{i}}{\delta_{i}}\right\}$$

*where*

(123) $$\begin{array}{c} \mathrm{diag}\left\{E\left(U^{T}X-U^{T}\hat{X}\right){\left(U^{T}X-U^{T}\hat{X}\right)}^{T}\right\}=\mathrm{diag}\left\{\delta_{1},\delta_{2},\ldots ,\delta_{n_{x}}\right\}. \end{array}$$

**Proof.**  By Theorem 3a,

(124) $$\begin{array}{cc} \hat{X}= & HX+\left(I-H\right)Q_{X,Y}Q_{Y}^{-1}Y+W \end{array}$$

(125) $$\begin{array}{cc} = & H\left(X-Q_{X,Y}Q_{Y}^{-1}Y\right)+Q_{X,Y}Q_{Y}^{-1}Y+W \end{array}$$

(126) $$\begin{array}{cc} ⟹ & \;\hat{X}-Q_{X,Y}Q_{Y}^{-1}Y=H\left(X-Q_{X,Y}Q_{Y}^{-1}Y\right)+W \end{array}$$

(127) $$\begin{array}{cc} ⟹ & \;\hat{X}=HX+W. \end{array}$$

The last equation establishes (115). By properties of conditional mutual information and the properties of optimal realization $\hat{X}$, the following equalities hold.

(128) $$\begin{array}{cc} I(X;\hat{X}|Y)= & I(X-Q_{X,Y}Q_{Y}^{-1}Y;\hat{X}-Q_{X,Y}Q_{Y}^{-1}Y|Y) \end{array}$$

(129) $$\begin{array}{cc} = & I(X;\hat{X}|Y),\;\mathrm{by}\;(112) \end{array}$$

(130) $$\begin{array}{cc} = & H\left(\hat{X}|Y\right)-H\left(\hat{X}|Y,X\right) \end{array}$$

(131) $$\begin{array}{cc} = & H\left(\hat{X}\right)-H\left(\hat{X}|Y,X\right),\;\mathrm{by}\;\mathrm{indep}.\mathrm{of}\;(X,W)\;\mathrm{and}\;Y \end{array}$$

(132) $$\begin{array}{cc} = & H\left(\hat{X}\right)-H\left(\hat{X}|X\right),\;\mathrm{by}\;\mathrm{indep}.\mathrm{of}\;W\;\mathrm{and}\;Y\;\mathrm{for}\;\mathrm{fixed}\;X \end{array}$$

(133) $$\begin{array}{cc} = & I(X;\hat{X}) \end{array}$$

(134) $$\begin{array}{cc} = & I(U^{T}X;U^{T}\hat{X}) \end{array}$$

(135) $$\begin{array}{cc} = & I({\bar{X}}_{1},{\bar{X}}_{2},\ldots ,{\bar{X}}_{n_{x}};{\hat{\bar{X}}}_{1},{\hat{\bar{X}}}_{2},\ldots ,{\hat{\bar{X}}}_{n_{x}}) \end{array}$$

(136) $$\begin{array}{cc} \geq & \sum_{t=1}^{n_{x}}I\left({\bar{X}}_{t};{\hat{\bar{X}}}_{t}\right),\;\mathrm{by}\;\mathrm{mutual}\;\mathrm{independence}\;\mathrm{of}\;{\bar{X}}_{t},t=1,2,\ldots ,n_{x}. \end{array}$$

Moreover, inequality (136) holds with equality if $\left({\bar{X}}_{t};{\hat{\bar{X}}}_{t}\right),t=1,2,\ldots ,n_{x}$ are jointly independent. The average distortion function is then given by

(137) $$\begin{array}{cc} E∥X-\hat{X}{∥}_{R^{n_{x}}}^{2} & =E∥X-\hat{X}-Q_{X,Y}Q_{Y}^{-1}Y+Q_{X,Y}Q_{Y}^{-1}Y{∥}_{R^{n_{x}}}^{2} \end{array}$$

(138) $$\begin{array}{cc} \; & =E∥X-\hat{X}{∥}_{R^{n_{x}}}^{2},\;by(112) \end{array}$$

(139) $$\begin{array}{cc} \; & =E∥U^{T}X-U^{T}\hat{X}{∥}_{R^{n_{x}}}^{2}=\mathrm{trace}\left(\Sigma_{\Delta }\right),\;byUU^{T}=I_{n_{x}}. \end{array}$$

By Corollary 1, if (105) holds, that is, $Q_{X|Y}≻0$ and $\Sigma_{\Delta }⪰0$ satisfy $Q_{X|Y}\Sigma_{\Delta }=\Sigma_{\Delta }Q_{X|Y}$ (i.e., commute), then (106)–(108) hold, and by (122) we obtain

(140) $$\begin{array}{cc} \hat{\bar{X}}\overset{▵}{=} & U^{T}\hat{X}=U^{T}HX+U^{T}W=U^{T}\hat{X}=U^{T}HUU^{T}X+U^{T}W \end{array}$$

(141) $$\begin{array}{cc} = & U^{T}HU\bar{X}+U^{T}W,\;U^{T}HU\;\mathrm{is}\;\mathrm{diagonal}\;\mathrm{and}\;U^{T}W\;\mathrm{has}\;\mathrm{indep}.\;\mathrm{components}. \end{array}$$

Hence, if (105) holds, then the lower bound in (136) holds with equality because $\left({\bar{X}}_{t};{\hat{\bar{X}}}_{t}\right),t\in Z_{n_{x}}$ are jointly independent. Moreover, if (105) holds, then from, say, (118), the expressions (122) and (123) are obtained. The above equations establish all claims. □

**Proposition 4.**  *Theorem 4 is correct.*

**Proof.**  By invoking Corollary 2, Theorem 7 and the convexity of $R_{X|Y}\left(\Delta_{X}\right)$ given by (122), then we arrive at the statements of Theorem 4, which completely characterize the RDF $R_{X|Y}\left(\Delta_{X}\right)$ and construct a realization of the optimal $\hat{X}$ that achieves it. □

Next, we discuss the degenerate case, when the statements of Theorems 3, 4 and 7 reduce to the RDF $R_{X}\left(\Delta_{X}\right)$ of a Gaussian RV *X* with square-error distortion function. We illustrate that the identified structural property of the realization matrices $\left\{\Sigma_{\Delta },Q_{X|Y},H,Q_{W}\right\}$ leads to to the well-known water-filling solution.

**Remark 3.**  *Degenerate case of Theorem 7 and realization $\hat{X}$ of Theorem 4a. Consider the characterization of the RDF $R_{X|Y}\left(\Delta_{X}\right)$ of Theorem 7, the realization of $\hat{X}$ Theorem 3a, Theorem 3, and assume X and Y are independent or Y generates the trivial information; i.e., the σ−algebra of Y is σ{Y}={Ω,∅} or C=0 in (15)–(18).*

*(a) By the definitions of $Q_{X,Y},Q_{X|Y}$ then*

(142) $$\begin{array}{c} Q_{X,Y}=0,\;Q_{X|Y}=Q_{X}. \end{array}$$

*Substituting (142) into the expressions of Theorem 7, the RDF $R_{X|Y}\left(\Delta_{X}\right)$ reduces to*

(143) $$\begin{array}{cc} R_{X|Y}\left(\Delta_{X}\right)= & R_{X}\left(\Delta_{X}\right)\overset{▵}{=}\underset{Q(\Delta_{X})}{\inf}I\left(X;\hat{X}\right) \end{array}$$

(144) $$\begin{array}{cc} = & \underset{Q^{m}\left(\Delta_{X}\right)}{\inf}\frac{1}{2}\log\left\{\det(Q_{X}\Sigma_{\Delta }^{-1})\right\}, \end{array}$$

*where*

(145) $$\begin{array}{cc} Q^{m}\left(\Delta_{X}\right)\overset{▵}{=} & \left\{\Sigma_{\Delta }⪰0:\;Q_{X}⪰\Sigma_{\Delta },\;\mathrm{trace}\left(\Sigma_{\Delta }\right)\leq \Delta_{X}\right\}, \end{array}$$

*and the optimal reproduction $\hat{X}$ reduces to*

(146) $$\begin{array}{cc} \hat{X}= & \left(I_{n_{x}}-\Sigma_{\Delta }Q_{X}^{-1}\right)X+W,\;Q_{X}⪰\Sigma_{\Delta }, \end{array}$$

(147) $$\begin{array}{cc} Q_{W}= & \left(I_{n_{x}}-\Sigma_{\Delta }Q_{X}^{-1}\right)\Sigma_{\Delta }⪰0. \end{array}$$

*Thus, $R_{X}\left(\Delta_{X}\right)$ is the well-known RDF of a multivariate memoryless Gaussian RV X with square-error distortion.*

*(b) For the RDF $R_{X}\left(\Delta_{X}\right)$ of part (a), it is known [24] that $\Sigma_{\Delta }$ and $Q_{X}$ have a spectral decomposition with respect to the same unitary matrix, that is,*

(148) $$\begin{array}{cc} \; & Q_{X}=U\Lambda_{X}U^{T},\;\Sigma_{\Delta }=U\Delta U^{T},\;UU^{T}=I, \end{array}$$

(149) $$\begin{array}{cc} \; & \Lambda_{X}=\mathrm{diag}\left\{\lambda_{X,1},\cdots ,\lambda_{X,n_{x}}\right\},\;\Delta =\mathrm{diag}\left\{\delta_{1},\cdots ,\delta_{n_{x}}\right\}, \end{array}$$

*where the entries of $\left(\Lambda_{X},\Delta \right)$ are in decreasing order.*

*Define*

(150) $$\begin{array}{c} X^{p}\overset{▵}{=}U^{T}X,\;{\hat{X}}^{p}\overset{▵}{=}U^{T}\hat{X},\;W^{p}\overset{▵}{=}U^{T}W. \end{array}$$

*Then, a parallel channel realization of the optimal reproduction ${\hat{X}}^{p}$ is obtained as follows:*

(151) $$\begin{array}{cc} \; & {\hat{X}}^{p}=HX^{p}+W^{p}, \end{array}$$

(152) $$\begin{array}{cc} \; & H=I_{n_{x}}-\Delta \Lambda_{X}^{-1}=\mathrm{diag}\left\{1-\frac{\delta_{1}}{\lambda_{X,1}},\cdots ,1-\frac{\delta_{n_{x}}}{\lambda_{X,n_{x}}}\right\}, \end{array}$$

(153) $$\begin{array}{cc} \; & Q_{W^{p}}=H\Delta =\mathrm{diag}\left\{\left(1-\frac{\delta_{1}}{\lambda_{X,1}}\right)\delta_{1},\cdots ,\left(1-\frac{\delta_{n_{x}}}{\lambda_{X,n_{x}}}\right)\delta_{n_{x}}\right\}. \end{array}$$

*The RDF $R_{X}\left(\Delta_{X}\right)$ is then computed from the reverse water-filling equations as follows.*

(154) $$R_{X}\left(\Delta_{X}\right)=\frac{1}{2}\sum_{i=1}^{n_{x}}\log\frac{\lambda_{X,i}}{\delta_{i}},$$

*where*

(155) $$\sum_{i=1}^{n_{x}}\delta_{i}=\Delta_{X},\;\delta_{i}=\left\{\begin{array}{ccc} \mu , & if & \mu <\lambda_{X,i}\\ \lambda_{X,i}, & if & \mu \geq \lambda_{X,i} \end{array}\right.$$

*and μ∈[0,∞) is a Lagrange multiplier (obtained from the Kuch–Tucker conditions).*

### 4.2. Side Information Only at Decoder

In general, when the side information is available only at the decoder, the achievable operational rate $R^{*}\left(\Delta_{X}\right)$ is greater than the achievable operational rate ${\bar{R}}_{1}\left(\Delta_{X}\right)$ when the side information is available to the encoder and the decoder [2]. By Remark 1, $\bar{R}\left(\Delta_{X}\right)\geq R_{X|Y}\left(\Delta_{X}\right)$, and equality holds if $I(X;Z|\hat{X},Y)=0$.

In view of the characterization of $R_{X|Y}\left(\Delta_{X}\right)$ and the realization of the optimal reproduction $\hat{X}$ of Theorem 3, which is presented in Figure 3, we observe that we can re-write (49) as follows.

(156) $$\begin{array}{cc} \hat{X}= & HX+\left(I_{n_{x}}-H\right)Q_{X,Y}Q_{Y}^{-1}Y+W, \end{array}$$

(157) $$\begin{array}{cc} = & \left(I_{n_{x}}-H\right)Q_{X,Y}Q_{Y}^{-1}Y+Z \end{array}$$

(158) $$\begin{array}{cc} = & f(Y,Z) \end{array}$$

(159) $$\begin{array}{cc} Z= & HX+W, \end{array}$$

(160) $$\begin{array}{cc} H= & I_{n_{x}}-\Sigma_{\Delta }Q_{X|Y}^{-1},\;Q_{W}=H\Sigma_{\Delta },\;\mathrm{defined}\;\mathrm{by}\;(51)–(63), \end{array}$$

(161) $$\begin{array}{cc} P_{Z|X,Y}= & P_{Z|X},\;\left(\hat{X},Y\right)\mathrm{uniquely}\;\mathrm{define}\;Z,\;\mathrm{which}\;\mathrm{implies}\;I\left(X;Z|\hat{X},Y\right)=0. \end{array}$$

**Proposition 5.**  *Theorem 5 is correct.*

**Proof.**  From the above realization of $\hat{X}=f\left(Y,Z\right)$, we have the following. (a) By Wyner, see Remark 1, then the inequalities (36) and (37) hold, and equalities hold if $I(X;Z|\hat{X},Y)=0$. That is, for any $\hat{X}=f\left(Y,Z\right)$, and by the properties of conditional mutual information, then

(162) $$\begin{array}{cc} I(X;Z|Y) & \overset{\left(\alpha \right)}{=}I\left(X;Z,\hat{X}|Y\right) \end{array}$$

(163) $$\begin{array}{cc} \; & \overset{\left(\beta \right)}{=}I\left(X;Z|\hat{X},Y\right)+I\left(X;\hat{X}|Y\right) \end{array}$$

(164) $$\begin{array}{cc} \; & \overset{\left(\gamma \right)}{\geq }I\left(X;\hat{X}|Y\right), \end{array}$$

where (α) is due to $\hat{X}=f\left(Y,Z\right)$, (β) is due to the chain rule of mutual information, and (γ) is due to $I(X;Z|\hat{X},Y)\geq 0$. Hence, (72) is obtained (as in Wyner [2] for a tuple of scalar jointly Gaussian RVs). (b) Equality holds in (164) if there exists an $\hat{X}=f\left(Y,Z\right)$ such that $I(X;Z|\hat{X},Y)=0$, and the average distortion is satisfied. Taking $\hat{X}=f\left(Y,Z\right)=\left(I_{n_{x}}-H\right)Q_{X,Y}Q_{Y}^{-1}Y+Z$, where Z=g(X,W) is specified by (156)–(160), then $I(X;Z|\hat{X},Y)=0$ and the average distortion is satisfied. Since the realization (156)–(160) is identical to the realization (73)–(76), then part (b) is also shown. (c) This follows directly from the optimal realization. □

## 5. Connection with Other Works and Simulations

In this section, we illustrate that for the special case of scalar-valued jointly Gaussian RVs (X,Y), our results reproduce Wyner’s [2] results. In addition, we show that the characterizations of the RDFs of the more general problems considered in [5,6] (i.e., where a noisy version of source is available at the encoder) do not reproduce Wyner’s [2] results. Finally, we present simulations.

### 5.1. Connection with Other Works

**Figure 4:**
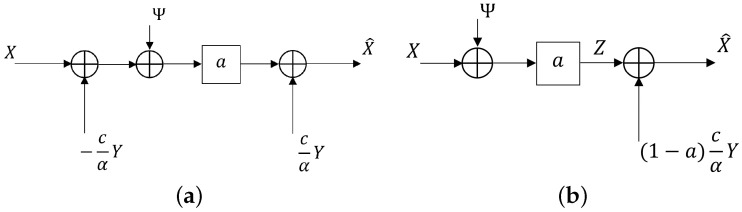
Wyner’s realizations of optimal reproductions for RDFs $R_{X|Y}\left(\Delta_{X}\right)$ and $\bar{R}\left(\Delta_{X}\right)$. (**a**) RDF $R_{X|Y}\left(\Delta_{X}\right)$: Wyner’s [2] optimal realization of $\hat{X}$ for RDF $R_{X|Y}\left(\Delta_{X}\right)$ of (165)–(168). (**b**) RDF $\bar{R}\left(\Delta_{X}\right)$: Wyner’s [2] optimal realization $\hat{X}=f\left(X,Z\right)$ for RDF $\bar{R}\left(\Delta_{X}\right)$ of (165)–(168).

**Remark 4.**  *The degenerate case to Wyner’s [2] optimal test channel realizations. Now, we verify that for the tuple of scalar-valued, jointly Gaussian RVs (X,Y), with square error distortion function specified below, our optimal realizations of $\hat{X}$ and closed form expressions for $R_{X|Y}\left(\Delta_{X}\right)$ and $\bar{R}\left(\Delta_{X}\right)$ are identical to Wyner’s [2] realizations and RDFs (see Figure 4). Let us define:*

(165) $$\begin{array}{cc} \; & X:\Omega \to X\overset{▵}{=}R,\;Y:\Omega \to Y\overset{▵}{=}R,\;\hat{X}:\Omega \to \hat{X}\overset{▵}{=}R, \end{array}$$

(166) $$\begin{array}{cc} \; & d_{X}\left(x,\hat{x}\right)={\left(x-\hat{x}\right)}^{2}, \end{array}$$

(167) $$\begin{array}{cc} \; & X\in N\left(0,\sigma_{X}^{2}\right),\;\sigma_{X}^{2}>0,\;Y=\alpha \left(X+U\right), \end{array}$$

(168) $$\begin{array}{cc} \; & U\in N\left(0,\sigma_{U}^{2}\right),\;\sigma_{U}^{2}>0,\;\alpha >0. \end{array}$$

*(a) RDF $R_{X|Y}\left(\Delta_{X}\right)$: By Theorem 4a applied to (165)–(168), we obtain*

(169) $$\begin{array}{cc} \; & Q_{X}=\sigma_{X}^{2},\;Q_{X,Y}=\alpha \sigma_{X}^{2},\;Q_{Y}=\sigma_{Y}^{2}=\alpha^{2}\sigma_{X}^{2}+\alpha^{2}\sigma_{U}^{2},\;Q_{X|Y}=c\sigma_{U}^{2},\;c\overset{▵}{=}\frac{\sigma_{X}^{2}}{\sigma_{X}^{2}+\sigma_{U}^{2}}, \end{array}$$

(170) $$\begin{array}{cc} \; & H=1-\Delta_{X}Q_{X|Y}^{-1}=\frac{c\sigma_{U}^{2}-d}{c\sigma_{U}^{2}}\equiv a,\;Q_{X,Y}Q_{Y}^{-1}=\frac{c}{\alpha },\;HQ_{X,Y}Q_{Y}^{-1}=\frac{ac}{\alpha }, \end{array}$$

(171) $$\begin{array}{cc} \; & W=H\Psi =a\Psi ,\;Q_{\Psi }=H^{-1}\Delta_{X}=\frac{\Delta_{X}}{a}=\frac{c\sigma_{U}^{2}\Delta_{X}}{c\sigma_{U}^{2}-\Delta_{X}},\;c\sigma_{U}^{2}-\Delta_{X}>0. \end{array}$$

*Moreover, by Theorem 4b the optimal reproduction $\hat{X}\in M_{0}\left(d\right)$ and $R_{X|Y}\left(d\right)$ are,*

(172) $$\begin{array}{cc} \; & \hat{X}=a\left(X-\frac{c}{\alpha }Y\right)+\frac{c}{\alpha }Y+a\Psi ,\;c\sigma_{U}^{2}-\Delta_{X}>0 \end{array}$$

(173) $$\begin{array}{cc} \; & R_{X|Y}\left(\Delta_{X}\right)=\left\{\begin{array}{cc} \frac{1}{2}\log\frac{c\sigma_{U}^{2}}{\Delta_{X}}, & 0<\Delta_{X}<c\sigma_{U}^{2}\\ 0, & \Delta_{X}\geq c\sigma_{U}^{2}. \end{array}\right. \end{array}$$

*This shows our realization of Figure 3 degenerates to Wyner’s [2] realization of Figure 4a.*

*(b) RDF $\bar{R}\left(\Delta_{X}\right)$: By Theorem 5b applied to (165)–(168), and using the calculations (169)–(172), we obtain*

(174) $$\begin{array}{cc} \; & \hat{X}=f\left(Y,Z\right)=\frac{c}{\alpha }\left(1-a\right)Y+Z\;by\;(172),(175), \end{array}$$

(175) $$\begin{array}{cc} \; & Z=a\left(X+\Psi \right),\;\left(a,\Psi \right)\;definedin\;(170),(171) \end{array}$$

(176) $$\begin{array}{cc} \; & \bar{R}\left(\Delta_{X}\right)=R_{X|Y}\left(\Delta_{X}\right)=\left(173\right)\;by\;evaluating\;I(X;Z)-I(Y;Z),\;using\;(4)\;and\;(175). \end{array}$$

*This shows our value of $\bar{R}\left(\Delta_{X}\right)$ and optimal realization $\hat{X}=f\left(Y,Z\right)$ reproduce Wyner’s optimal realization and the value of $\bar{R}\left(\Delta_{X}\right)$ given in [2] (i.e., Figure 4b).*

In the following Remark, we show that, when S=X-a.s., the realization of the auxiliary RV *Z*, which is used in the proofs in [5,6] to show the converse coding theorem does not coincide with Wyner’s realization [2]. Also, their realizations do not reproduce Wyner’s RDF (this observation is verified for modified realization given in the correction note without proof in https://tiangroup.engr.tamu.edu/publications/ (accessed on 3 January 2024)). The deficiency of the realizations in [5,6] to show the converse was first pointed out in [7], using an alternative proof.

**Remark 5.**  *Optimal test channel realization of [5,6]*

*(a) The derivation of [[5], Theorem 4], uses the following representation of RVs (see [[5], Equation (4)] adopted to our notation using (19)):*

$$\begin{array}{c} X=\left(K_{xs}K_{sy}+K_{xy}\right)Y+K_{xs}N_{1}+N_{2},\;\;S=K_{sy}Y+N_{1}, \end{array}$$

*where $N_{1}$ and $N_{2}$ are independent Gaussian RVs with zero mean, $N_{1}$ is independent Y and $N_{2}$ is independent of (S,Y).*

*To reduce [5,6] to the Wyner and Ziv RDF, we set X=S−a.s., which then implies, $K_{xs}=I,\;N_{2}=0-a.s$ and $K_{xy}=0$. According to the derivation of the converse [[5], Theorem 4] (see [[5], 3 lines above Equation (32)] using our notation), the optimal realization of the auxiliary RV $Z_{T}$ used to achieve the RDF is*

(177) $$\begin{array}{c} Z_{T}=U^{T}X+N_{3}, \end{array}$$

*where $Q_{X|Y}=U\mathrm{diag}\left(\lambda_{1},\cdots ,\lambda_{n}\right)U^{T}$ and U is a unitary matrix, $N_{3}\in N\left(0,Q_{N_{3}}\right)$ such that $Q_{N_{3}}$ is a diagonal covariance matrix, with elements given by (for the value of $\sigma_{3,i}^{2}$, we considered the one given in the correction note in https://tiangroup.engr.tamu.edu/publications/ (accessed on 3 January 2024) (although no derivation is given), where it is stated that $\sigma_{3,i}^{2}$ that appeared in the derivation [[5], proof of theorem 4] should be multiplied by $\lambda_{i}$),*

(178) $$\begin{array}{c} \sigma_{3,i}^{2}=\frac{\min(\lambda_{i},\delta_{i})}{\lambda_{i}-\min\left(\lambda_{i},\delta_{i}\right)}\lambda_{i},\;\sum_{i=1}^{n}\min\left(\lambda_{i},\delta_{i}\right)=\Delta_{X}. \end{array}$$

*(b) It is easy to verify that the above realization of $Z_{T}$ that uses the correction of footnote 6 is precisely the realization given in [[6], Theorem 3A].*

*(c) Special Case: For scalar-valued RVs the auxiliary RV $Z_{T}$ reduces to*

(179) $$\begin{array}{c} Z_{T}=X+N_{3},\;N_{3}\in N\left(0,\frac{\Delta_{X}Q_{X|Y}}{Q_{X|Y}-\Delta_{X}}\right),\;Q_{X|Y}>\Delta_{X} \end{array}$$

*Now, we examine whether the realization (179) corresponds to Wyner’s realization and induces Wyner’s RDF. Recall that the Wyner’s [2] RDF, denoted by $R_{X;Z|Y}\left(\Delta_{X}\right)$ and corresponding to auxiliary RV Z, is*

(180) $$\begin{array}{cc} \; & Z=HX+W,\;H=\frac{Q_{X|Y}-\Delta_{X}}{Q_{X|Y}},\;W\in N\left(0,H\Delta_{X}\right), \end{array}$$

(181) $$\begin{array}{cc} \; & R_{X;Z|Y}\left(\Delta_{X}\right)=I\left(X;Z|Y\right)=\frac{1}{2}\log\left(\frac{Q_{X|Y}}{\Delta_{X}}\right),\;\Delta_{X}\leq Q_{X|Y}. \end{array}$$

*Clearly, the two realizations (179) and (180) are different. Let ${\hat{R}}_{X;Z_{T}|Y}\left(\Delta_{X}\right)$ denote the RDF corresponding to the realization $Z_{T}$. Then ${\hat{R}}_{X;Z_{T}|Y}\left(\Delta_{X}\right)$ can be computed using $I\left(X;Z_{T}|Y\right)=I\left(X;Z_{T}\right)-I\left(Y;Z_{T}\right)=-H\left(Z_{T}|X\right)+H\left(Z_{T}|Y\right)$ where H(·|·) denotes the conditional differential entropy. Then, by using*

(182) $$\begin{array}{cc} \; & Q_{Z_{T}|X}=Q_{N_{3}}=\frac{\Delta_{X}Q_{X|Y}}{Q_{X|Y}-\Delta_{X}}, \end{array}$$

(183) $$\begin{array}{cc} \; & Q_{Z_{T}|Y}=Q_{N_{3}}+Q_{X|Y}. \end{array}$$

*it is straightforward to show that*

(184) $$\begin{array}{cc} {\hat{R}}_{X;Z_{T}|Y}\left(\Delta_{X}\right)= & -H\left(Z_{T}|X\right)+H\left(Z_{T}|Y\right) \end{array}$$

(185) $$\begin{array}{cc} = & -\frac{1}{2}\log\left(2\pi e\frac{\Delta_{X}Q_{X|Y}}{Q_{X|Y}-\Delta_{X}}\right)+\frac{1}{2}\log\left(2\pi e\frac{Q_{X|Y}^{2}}{Q_{X|Y}-\Delta_{X}}\right),\;\Delta_{X}<Q_{X|Y} \end{array}$$

*However, we note that (i) unlike Wyner’s RDF given in (181), which gives $R_{X;Z|Y}\left(\Delta_{X}\right)=0$ at $\Delta_{X}=Q_{X|Y}$, the corresponding ${\hat{R}}_{X;Z_{T}|Y}\left(\Delta_{X}\right)=-\infty +\infty$ at $\Delta_{X}=Q_{X|Y}$, and (ii) Wyner’s test channel realization is Z=HX+W,$H=\frac{Q_{X|Y}-\Delta_{X}}{Q_{X|Y}}$ and $W\in N(0,H\Delta_{X})$, which is different from the test channel realization in (179). In particular, if $Q_{X|Y}=\Delta_{X}$, then H=0⇒W∈N(0,0) and Z=0−a.s. On the other hand, for the test channel in (179), if $Q_{X|Y}=\Delta_{X}$, then $N_{3}\in N\left(0,+\infty \right)$, and thus the variance of $Z_{T}$ in (179) is not zero.*

*Further, in Proposition 6, we prove that for the multi-dimensional source, the test channel realization in (179) does not achieve the RDF when water-filling is active, i.e., when at least one component of the source is not reproduced.*

*(d) Special Case Classical RDF: The classical RDF is obtained as a special case if we assume X and Y are independent or Y generates the trivial information {Ω,∅}; i.e., Y is nonrandom. Clearly, in this case, the RDF ${\hat{R}}_{S;Z_{T}|Y}\left(\Delta_{X}\right)$ should degenerate to the classical RDF of the source X, i.e., $R_{X}\left(\Delta_{X}\right)$, and it should be that $\hat{X}=Z_{T}$. However, for this case, (179) gives $Q_{Z_{T}}=Q_{X}+\frac{\Delta_{X}Q_{X}}{Q_{X}-\Delta_{X}}=\frac{Q_{X}^{2}}{Q_{X}-\Delta_{X}}$, which is fundamentally different from Wyner’s degenerate, and correct values $Q_{\hat{X}}=Q_{Z}=\max\left\{0,Q_{X}-\Delta_{X}\right\}$.*

**Proposition 6.**  *When S=X-a.s., Wyner’s [2] auxiliary RV Z and the auxiliary RV $Z_{T}$ given in (177) i.e., the degenerate case of [5,6] (with the correction of footnote 6), are not related by an invertible function. As a result, the computed RDF based on the two realizations are different.*

**Proof.**  Recall that, if the two auxiliary RVs $Z_{T}$ and *Z* are not related by an invertible function, i.e., $Z=f(Z_{T})$, where f(·) is invertible and both *f* and its inverse are measurable, then $I\left(X;Z_{T}\right)-I\left(Y;Z_{T}\right)\neq I\left(X;Z\right)-I\left(Y;Z\right)$. It was shown earlier in this paper (and also in [7]) that for the multivariate Wyner’s RDF, the auxiliary RV takes the form

(186) $$\begin{array}{c} Z=HX+W,\;W\in N(0,Q_{W}), \end{array}$$

where $Q_{W}=H\Sigma_{\Delta }=U\mathrm{diag}\left(\sigma_{w,1}^{2},\cdots ,\sigma_{w,n}^{2}\right)U^{T}$, $\Sigma_{\Delta }=U\mathrm{diag}\left(\delta_{1},\cdots ,\delta_{n}\right)U^{T}$, $H=I-Q_{X|Y}^{-1}\Sigma_{\Delta }=U\mathrm{diag}\left(h_{1},\cdots ,h_{n}\right)U^{T}$ and $Q_{X|Y}=U\mathrm{diag}\left(\lambda_{1},\cdots ,\lambda_{n}\right)U^{T}$, where *U* is a unitary matrix. The eigenvalues $\sigma_{w,i}^{2}$ and $h_{i}$ are given by

(187) $$\begin{array}{cc} \; & \sigma_{w,i}^{2}=\frac{\min\left(\lambda_{i},\delta_{i}\right)\left(\lambda_{i}-\min\left(\lambda_{i},\delta_{i}\right)\right)}{\lambda_{i}},\;\; \end{array}$$

(188) $$\begin{array}{cc} \; & h_{i}=\frac{\lambda_{i}-\min\left(\lambda_{i},\delta_{i}\right)}{\lambda_{i}}, \end{array}$$

where $\sum_{i=1}^{n}\min\left(\lambda_{i},\delta_{i}\right)=\Delta_{X}$. Hence, Equations (186), (187), and (188), imply that if $\sigma_{w,i}^{2}=0$ then $h_{i}=0$, and vice versa. Such zero values correspond to compression with water-filling. On the other hand, from (177) and (178), if water-filling is active, then $\sigma_{3,i}^{2}=\frac{\lambda_{i}^{2}}{\lambda_{i}-\lambda_{i}}$. Moreover, by comparing Equations (187) with (178) and (188) with (177), it is straightforward to show that f(·)=HU. If HU is not an invertible matrix for all values of the distortion $\Delta_{X}$, then $I\left(X;Z_{T}\right)-I\left(Y;Z_{T}\right)\neq I\left(X;Z\right)-I\left(Y;Z\right)$.

By (188) it is easy to show that if $\min\left(\lambda_{i},\delta_{i}\right)=\lambda_{i}$, HU is not invertible. This implies $I\left(X;Z_{T}\right)-I\left(Y;Z_{T}\right)\neq I\left(X;Z\right)-I\left(Y;Z\right)$. □

### 5.2. Simulations

In this section, we provide an example to show the gap between the classical rate distortion $R_{X}\left(\Delta_{X}\right)$ defined in (154), the conditional distortion function $R_{X|Y}\left(\Delta_{X}\right)$ (69), and to verify the validity of Gray’s lower bound (8). Note that in Theorem 5 it is shown that $R_{X|Y}\left(\Delta_{X}\right)=\bar{R}\left(\Delta_{X}\right)$, and hence the plot for $\bar{R}\left(\Delta_{X}\right)$ is omitted. For the evaluation, we pick a joint covariance matrix (11) given by

$$\begin{array}{cc} \; & Q_{\left(X,Y\right)}=\left[\begin{array}{cccc} 2.5000 & 1.1250 & 0.4750 & 0.6125\\ 1.1250 & 0.8125 & 0.2750 & 0.3063\\ 0.4750 & 0.2750 & 0.1525 & 0.1625\\ 0.6125 & 0.3063 & 0.1625 & 0.2031 \end{array}\right],\;X:\Omega \to R^{2},\;Y:\Omega \to R^{2}. \end{array}$$

In order to compute the rates, we first have to find $Q_{X},Q_{Y},Q_{XY}$ and $Q_{X|Y}$. From the definition of $Q_{\left(X,Y\right)}$ given in (11), it is easy to see that the covariance of *X*, *Y*, and the joint covariance of *X* and *Y* are equal to

$$\begin{array}{c} Q_{X}=\left[\begin{array}{cc} 2.5000 & 1.1250\\ 1.1250 & 0.8125 \end{array}\right],\;\;Q_{Y}=\left[\begin{array}{cc} 0.1525 & 0.1625\\ 0.1625 & 0.2031 \end{array}\right],\;\;Q_{XY}=\left[\begin{array}{cc} 0.4750 & 0.6125\\ 0.2750 & 0.3063 \end{array}\right]. \end{array}$$

Then, the conditional covariance $Q_{X|Y}$, which appears in $R_{X|Y}\left(\Delta_{X}\right)$, can be computed from (27). Using Singular Value Decomposition (SVD), we can calculate the eigenvalues of $Q_{X|Y}$. For this case, the eigenvalues of the conditional covariance are {0.7538,0.2}. Similarly, the eigenvalues of $Q_{X}$ can be determined. Finally, the eigenvalues of $Q_{X}$ and $Q_{X|Y}$ are passed to the water-filling to compute the $R_{X}\left(\Delta_{X}\right)$ and $R_{X|Y}\left(\Delta_{X}\right)$, respectively.

**Figure 5:**
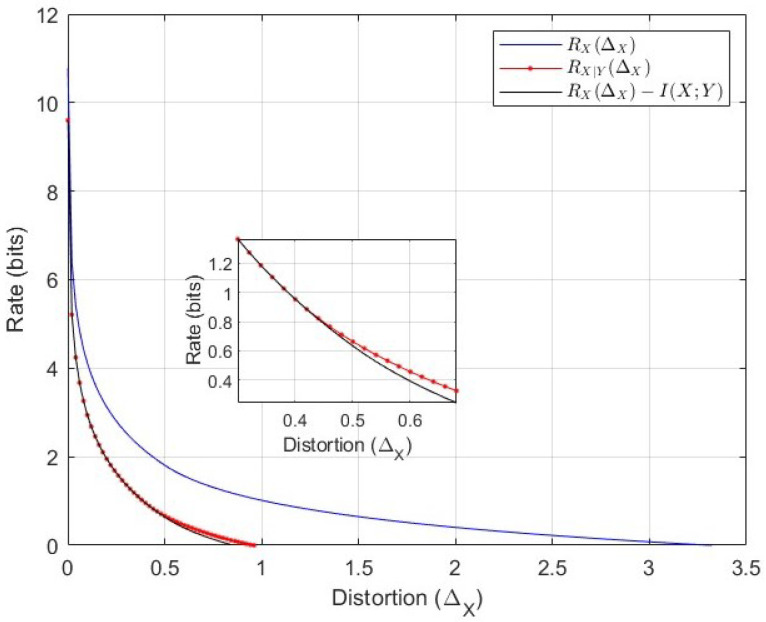
Comparison of classical RDF, $R_{X}\left(\Delta_{X}\right)$, conditional RDF $R_{X|Y}\left(\Delta_{X}\right)=\bar{R}\left(\Delta_{X}\right)$, and Gray’s lower bound $R_{X}\left(\Delta_{X}\right)-I\left(X;Y\right)$ (solid green line).

The classical rate distortion, the conditional RDF, and the Gray’s lower bound for the joint covariance above are illustrated in Figure 5. It is clear that $R_{X|Y}\left(\Delta_{X}\right)$ is smaller, and as the distortion $\Delta_{X}$ increases, the gap between the classical and conditional RDF becomes larger. Gray’s lower bound is achievable for some positive distortion values, as provided in (71), i.e., for $\Delta_{X}\in \left\{\Delta_{X}\in \left[0,\infty \right):\Delta_{X}\leq n_{x}\lambda_{n_{x}}\right\}$. Recall that the set of eigenvalues of $Q_{X|Y}$ is {0.7538,0.2}, and the lower bound is achievable for $\Delta_{X}\leq 2\cdot 0.2=0.4$; i.e., for these values $R_{X|Y}\left(\Delta_{X}\right)=R_{X}\left(\Delta_{X}\right)-I\left(X;Y\right)$.

## 6. Conclusions

We derived nontrivial structural properties of the optimal test channel realizations that achieve the optimal test channel distributions of the characterizations of RDFs for a tuple of multivariate jointly independent and identically distributed Gaussian random variables with mean-square error fidelity for two cases. Initially, the side information was available at the encoder and decoder, and then it was only available at the decoder. Using the realizations of the optimal test channels, we showed that when the side information is known to the encoder and the decoder, it does not achieve a better compression compared to when side information is only known to the decoder.

## Data Availability

Data are contained within the article.

## Appendix A

### Appendix A.1. Proof of Lemma 1

(a) By the chain rule of mutual information,

(A1) $$\begin{array}{cc} I(X;\hat{X},Y)= & I\left(X;Y|\hat{X}\right)+I\left(X;\hat{X}\right) \end{array}$$

(A2) $$\begin{array}{cc} = & I\left(X;\hat{X}|Y\right)+I\left(X;Y\right) \end{array}$$

Since $I(X;Y|\hat{X})\geq 0$, then from the above it follows

(A3) $$\begin{array}{cc} I(X;\hat{X})\leq & I\left(X;\hat{X}|Y\right)+I\left(X;Y\right) \end{array}$$

(A4) $$\begin{array}{cc} I(X;\hat{X}|Y)\geq & I\left(X;\hat{X}\right)-I\left(X;Y\right) \end{array}$$

The above shows (40). However, the inequality holds with equality if and only if $I(X;Y|\hat{X})=0$, and this quantity is zero if and only if $P_{X|\hat{X},Y}=P_{X|\hat{X}}$. Alternatively, we note the following:

$$\begin{array}{cc} I(X;\hat{X}|Y) & =E\left[\log\frac{P_{X|\hat{X},Y}}{P_{X|Y}}\right]\\ \; & =E\left[\log\frac{P_{X|\hat{X},Y}}{P_{X|Y}}\frac{P_{X}}{P_{X}}\right]\\ \; & =E\left[\log\frac{P_{X|\hat{X},Y}}{P_{X}}-\log\frac{P_{X|Y}}{P_{X}}\right]\\ \; & =E\left[\log\frac{P_{X|\hat{X}}}{P_{X}}-\log\frac{P_{X|Y}}{P_{X}}\right],\;\mathrm{if}\;\mathrm{and}\;\mathrm{only}\;\mathrm{if}P_{X|\hat{X},Y}=P_{X|\hat{X}}. \end{array}$$

This completes the statement of equality of (40); i.e., it establishes equality (41). (b) Consider a test channel $P_{X|\hat{X},Y}$ such that $E\{||X-\hat{X}|{|}_{R^{n_{x}}}^{2}\leq \Delta_{X}$, i.e., $\hat{X}\in M_{0}\left(\Delta_{X}\right)$, and such that $P_{X|\hat{X},Y}=P_{X|\hat{X}}$, for $\Delta_{X}\in D_{C}\left(X|Y\right)\subseteq \left[0,\infty \right)$. By (41) taking the infimum of both sides over $\hat{X}\in M_{0}\left(\Delta_{X}\right)$ such that $P_{X|\hat{X},Y}=P_{X|\hat{X}}$, then (43) is obtained on a nontrivial surface, i.e., $\Delta_{X}\in D_{C}\left(X|Y\right)$, which exists due to continuity and convexity of $R_{X}\left(\Delta_{X}\right)$ for $\Delta_{X}\in \left(0,\infty \right)$. This completes the proof.

### Appendix A.2. Proof of Theorem 2

(*a*) (1) By properties of conditional mutual information [18],

(A5) $$\begin{array}{cc} I\left(X;\hat{X}|Y\right)\overset{\left(\alpha \right)}{=} & I(X;\hat{X},{\bar{X}}^{cm}|Y) \end{array}$$

(A6) $$\begin{array}{cc} \overset{\left(\beta \right)}{=} & I\left(X;\hat{X}|{\bar{X}}^{cm},Y\right)+I\left(X;{\bar{X}}^{cm}|Y\right) \end{array}$$

(A7) $$\begin{array}{cc} \overset{\left(\gamma \right)}{\geq } & I(X;{\bar{X}}^{cm}|Y) \end{array}$$

where (α) is due to ${\bar{X}}^{cm}$ being a function of $\left(Y,\hat{X}\right)$, and a well-known property of the mutual information [18] (β) is due to the chain rule of mutual information [18], and (γ) is due to $I(X;\hat{X}|{\bar{X}}^{cm},Y)\geq 0$. Hence, inequality (45) is shown. (2) If (i) holds, i.e., $\hat{X}={\bar{X}}^{cm}$- a.s, then $I(X;\hat{X}|{\bar{X}}^{cm},Y)=0$, and hence the inequality (45) becomes an equality. If (ii) holds, since for fixed y∈Y the function $e\left(y,\cdot \right):\hat{X}\to X$, $e\left(y,\hat{x}\right)={\bar{x}}^{cm}$ uniquely defines $\hat{x}$, then $I(X;\hat{X}|{\bar{X}}^{cm},Y)=0$, and the inequality (45) becomes an equality.

(*b*) The inequality (48) is well known due to the orthogonal projection theorem.

### Appendix A.3. Proof of Theorem 7

Consider the realization (88). We identify the triple $\left(H,G,Q_{W}\right)$ such that (84) or (87) hold; i.e., we characterize the set $M_{0}^{G,o}\left(\Delta_{X}\right)$.

Case (i). $\mathrm{cov}(\hat{X},\hat{X}|Y)≻0$, that is, $\mathrm{rank}\left(Q_{\hat{X}|Y}\right)=n_{x}$. By Theorem 6, Case (i), we seek the triple $\left(H,G,Q_{W}\right)$ such that (84) holds, i.e., ${\bar{X}}^{cm}=\hat{X}-a.s$. Recall that Conditions 1 and 2 of Theorem 6 are sufficient for $\hat{X}={\bar{X}}^{cm}$.

*Condition 1, i.e.,* (85). The left-hand side part of (85) is given by (this follows from mean-square estimation theory, or an application of (26) with G={Ω,∅})

(A8) $$\begin{array}{cc} E\left(X|Y\right)= & E\left(X\right)+\mathrm{cov}\left(X,Y\right)\mathrm{cov}{\left(Y,Y\right)}^{-1}\left(Y-E\left(Y\right)\right)\\ = & \mathrm{cov}\left(X,Y\right)\mathrm{cov}{\left(Y,Y\right)}^{-1}Y \end{array}$$

(A9) $$\begin{array}{cc} = & Q_{X,Y}Q_{Y}^{-1}Y \end{array}$$

(A10) $$\begin{array}{cc} = & Q_{X}C^{T}Q_{Y}^{-1}Y\;\mathrm{by}\;\mathrm{model}\;(15)–(18). \end{array}$$

Similarly, the right hand side of (85) is given by

(A11) $$\begin{array}{cc} E\left(\hat{X}|Y\right)= & E\left(\hat{X}\right)+\mathrm{cov}\left(\hat{X},Y\right)\mathrm{cov}{\left(Y,Y\right)}^{-1}\left(Y-E\left(Y\right)\right)\\ = & \mathrm{cov}\left(\hat{X},Y\right)\mathrm{cov}{\left(Y,Y\right)}^{-1}Y \end{array}$$

(A12) $$\begin{array}{cc} = & \left(HQ_{X,Y}+GQ_{Y}\right)Q_{Y}^{-1}Y \end{array}$$

(A13) $$\begin{array}{cc} = & \left(HQ_{X}C^{T}+GQ_{Y}\right)Q_{Y}^{-1}Y\;\mathrm{by}\;(15)–(18) \end{array}$$

Equating (A9) and (A12), then

(A14) $$\begin{array}{cc} \; & E\left(X|Y\right)=E\left(\hat{X}|Y\right) \end{array}$$

(A15) $$\begin{array}{cc} \; & ⟹\;Q_{X,Y}Q_{Y}^{-1}Y=\left(HQ_{X,Y}+GQ_{Y}\right)Q_{Y}^{-1}Y\;\mathrm{by}\;(A12) \end{array}$$

(A16) $$\begin{array}{cc} \; & ⟹\;G=\left(I-H\right)Q_{X,Y}Q_{Y}^{-1} \end{array}$$

(A17) $$\begin{array}{cc} \; & ⟹\;G=\left(I-H\right)Q_{X}C^{T}Q_{Y}^{-1}\;\mathrm{by}\;(15)–(18). \end{array}$$

Hence, *G* is obtained, and the reproduction is represented by

(A18) $$\begin{array}{cc} \; & \hat{X}=HX+\left(I-H\right)Q_{X,Y}Q_{Y}^{-1}Y+W, \end{array}$$

(A19) $$\begin{array}{cc} \; & E\left(\hat{X}|Y\right)=Q_{X,Y}Q_{Y}^{-1}Y=E\left(X|Y\right), \end{array}$$

(A20) $$\begin{array}{cc} \; & \hat{X}-E\left(\hat{X}|Y\right)=HX-HQ_{X,Y}Q_{Y}^{-1}Y+W. \end{array}$$

*Condition 2, i.e.,* (86). To apply (86), the following calculations are needed.

(A21) $$\begin{array}{cc} Q_{X|Y} & \overset{▵}{=}\mathrm{cov}\left(X,X|Y\right)\\ \; & =E\left\{\left(X-E\left(X|Y\right)\right){\left(X-E\left(X|Y\right)\right)}^{T}\right\} \end{array}$$

(A22) $$\begin{array}{cc} \; & =Q_{X}-Q_{X,Y}Q_{Y}^{-1}Q_{X,Y}^{T} \end{array}$$

(A23) $$\begin{array}{cc} \; & =Q_{X}-Q_{X}C^{T}Q_{Y}^{-1}CQ_{X}\;\mathrm{by}\;(15)-(18) \end{array}$$

$$\begin{array}{cc} \mathrm{cov}(X,\hat{X}|Y) & \overset{▵}{=}E\left\{\left(X-E\left(X|Y\right)\right){\left(\hat{X}-E\left(\hat{X}|Y\right)\right)}^{T}\right\} \end{array}$$

(A24) $$\begin{array}{cc} \; & =E\left\{\left(X-E\left(X|Y\right)\right){\left(\hat{X}-E\left(X|Y\right)\right)}^{T}\right\}\;\mathrm{by}\;(A19) \end{array}$$

(A25) $$\begin{array}{cc} \; & =E\left\{\left(X-E\left(X|Y\right)\right){\left(\hat{X}\right)}^{T}\right\}\;\mathrm{by}\;\mathrm{orthogonality} \end{array}$$

(A26) $$\begin{array}{cc} \; & =Q_{X}H^{T}-Q_{X,Y}Q_{Y}^{-1}Q_{Y,X}H^{T}\;\mathrm{by}\;(A18),(A19) \end{array}$$

(A27) $$\begin{array}{cc} \; & =Q_{X}H^{T}-Q_{X}C^{T}Q_{Y}^{-1}CQ_{X}H^{T}\;\mathrm{by}\;(15)–(18)\\ \; & =\left(Q_{X}-Q_{X}C^{T}Q_{Y}^{-1}CQ_{X}\right)H^{T} \end{array}$$

(A28) $$\begin{array}{cc} \; & =Q_{X|Y}H^{T}. \end{array}$$

(A29) $$\begin{array}{cc} \mathrm{cov}(\hat{X},\hat{X}|Y) & \overset{▵}{=}E\left\{\left(\hat{X}-E\left(\hat{X}|Y\right)\right){\left(\hat{X}-E\left(\hat{X}|Y\right)\right)}^{T}\right\}\\ \; & =HQ_{X}H^{T}+Q_{W}-HQ_{X,Y}Q_{Y}^{-1}Q_{Y,X}H^{T}\;\mathrm{by}\;(A20) \end{array}$$

(A30) $$\begin{array}{cc} \; & =HQ_{X}H^{T}+Q_{W}-HQ_{X}C^{T}Q_{Y}^{-1}CQ_{X}H^{T}\;\mathrm{by}\;(15)–(18)\\ \; & =H\left(Q_{X}-Q_{X}C^{T}Q_{Y}^{-1}CQ_{X}\right)H^{T}+Q_{W} \end{array}$$

(A31) $$\begin{array}{cc} \; & =HQ_{X|Y}H^{T}+Q_{W}. \end{array}$$

By Condition 2 and (A28) and (A31),

(A32) $$\begin{array}{cc} \; & \mathrm{cov}\left(X,\hat{X}|Y\right)\mathrm{cov}{\left(\hat{X},\hat{X}|Y\right)}^{-1}=I_{n_{x}}\\ \; & ⟹\;Q_{X|Y}H^{T}{\left(HQ_{X|Y}H^{T}+Q_{W}\right)}^{-1}=I_{n_{x}} \end{array}$$

(A33) $$\begin{array}{cc} \; & ⟹\;Q_{W}=Q_{X|Y}H^{T}-HQ_{X|Y}H^{T} \end{array}$$

(A34) $$\begin{array}{cc} \; & ⟹\;Q_{W}=\left(I_{n_{x}}-H\right)Q_{X|Y}H^{T}. \end{array}$$

It remains to show $Q_{W}=Q_{W}^{T}$. This will follow shortly by identifying the equation for *H* as follows. Conditions 1 and 2 imply

(A35) $$\begin{array}{cc} \Sigma_{\Delta }= & \mathrm{cov}(X,X|Y,\hat{X}) \end{array}$$

(A36) $$\begin{array}{cc} = & \mathrm{cov}\left(X,X|Y\right)-\mathrm{cov}\left(X,\hat{X}|Y\right)\mathrm{cov}{\left(\hat{X},\hat{X}|Y\right)}^{-1}\mathrm{cov}{\left(X,\hat{X}|Y\right)}^{T},\mathrm{mboxby}\;\mathrm{Propostion}\;1,\;(26) \end{array}$$

(A37) $$\begin{array}{cc} = & \mathrm{cov}\left(X,X|Y\right)-\mathrm{cov}{\left(X,\hat{X}|Y\right)}^{T},\;\mathrm{by}\;(86) \end{array}$$

(A38) $$\begin{array}{cc} = & Q_{X|Y}-HQ_{X|Y},\;\mathrm{by}\;(A28). \end{array}$$

(A39) $$\begin{array}{cc} \; & ⟹\;HQ_{X|Y}=Q_{X|Y}-\Sigma_{\Delta } \end{array}$$

(A40) $$\begin{array}{cc} \; & ⟹\;H=I-\Sigma_{\Delta }Q_{X|Y}^{-1} \end{array}$$

By (A39), it then follows from (A33) that $Q_{W}=Q_{W}^{T}$. From the specification of *G* the equation of $Q_{W}$ given by (A33) and (A34) and $HQ_{X|Y},H$ given by (A39), and (A40) then follows the realization of Theorem 4.(a) for the case $Q_{X|Y}-\Sigma_{\Delta }≻0$. Properties (58)–(61) are easily verified.

Case (ii). $\mathrm{cov}(\hat{X},\hat{X}|Y)⪰0$ but not $\mathrm{cov}(\hat{X},\hat{X}|Y)≻0$, that is, $\mathrm{rank}\left(Q_{\hat{X}|Y}\right)=n_{1}<n_{x}$. We can verify that the stated realization in Theorem 4.(a) is such that Condition (87) holds. By (83) and the above calculations, we have

(A41) $$\begin{array}{cc} {\bar{X}}^{cm}=e^{G}\left(Y,\hat{X}\right)= & E\left(X|Y\right)+\mathrm{cov}\left(X,\hat{X}|Y\right){\left\{\mathrm{cov}(\hat{X},\hat{X}|Y)\right\}}^{†}\left(\hat{X}-E\left(\hat{X}|Y\right)\right) \end{array}$$

(A42) $$\begin{array}{cc} = & E\left(X|Y\right)+\left(Q_{X|Y}-\Sigma_{\Delta }\right){\left(Q_{X|Y}-\Sigma_{\Delta }\right)}^{†}\left(\hat{X}-E\left(\hat{X}|Y\right)\right). \end{array}$$

Since $Q_{\hat{X}|Y}=Q_{X|Y}-\Sigma_{\Delta }$, $E\left\{\left(\hat{X}-E\left(\hat{X}|Y\right)\right){\left(\hat{X}-E\left(\hat{X}|Y\right)\right)}^{T}\right\}=Q_{X|Y}-\Sigma_{\Delta }$, and $\mathrm{rank}\left(L\right)=n_{1}$, where $L=\left(Q_{X|Y}-\Sigma_{\Delta }\right){\left(Q_{X|Y}-\Sigma_{\Delta }\right)}^{†}$, then an application of Proposition 3, implies that Condition (87) holds. Thus, we have established Theorem 3.(a) and the properties stated under Theorem 4.(a). (i)–(iv). Finally, (96)–(101) are obtained from the realization, and hence Theorem 3.(b) is achievable.

### Appendix A.4. Proof of Corollary 1

(a) This part is a special case of a related statement in [22]. However, we include it for completeness. By linear algebra [21], given two matrices $A\in S_{+}^{k\times k},B\in S_{+}^{k\times k}$, then the following statements are equivalent: (1) AB is normal and (2) AB⪰0, where AB normal means $\left(AB\right){\left(AB\right)}^{T}={\left(AB\right)}^{T}\left(AB\right)$. Note that AB is normal if and only if AB=BA; i.e., they commute. Let $A=U_{A}D_{A}U_{A}^{T},B=U_{B}D_{B}U_{B}^{T},U_{A}U_{A}^{T}=I_{k},U_{B}U_{B}^{T}=I_{k}$,; i.e., there exists a spectral representation of A,B in terms of unitary matrices $U_{A},U_{B}$ and diagonal matrices $D_{A},D_{B}$. Then, AB⪰0 if and only if the matrices *A* and *B* commute; i.e., AB=BA, and *A* and *B* commute if and only if $U_{A}=U_{B}$.

Suppose (105) holds. Letting $A=Q_{X|Y},B=\Sigma_{\Delta }$, then $A=U_{A}D_{A}U_{A}^{T},$ $B=U_{B}D_{B}U_{B}^{T},$$U_{A}U_{A}^{T}=I_{n_{x}},U_{B}U_{B}^{T}=I_{n_{x}},U_{A}=U_{B}$. Since $Q_{X|Y}^{-1}=A^{-1}=U_{A}D_{A}^{-1}U_{A}^{T},$ then $\Sigma_{\Delta }Q_{X|Y}^{-1}=Q_{X|Y}^{-1}\Sigma_{\Delta }$; i.e., they commute. Hence,

(A43) $$\begin{array}{cc} H_{t}^{T}= & (I_{n_{x}}-{\left(\Sigma_{\Delta }Q_{X|Y}^{-1}\right)}^{T}=I_{n_{x}}-{\left(Q_{X|Y}^{-1}\right)}^{T}\Sigma_{\Delta }^{T}=I_{n_{x}}-Q_{X|Y}^{-1}\Sigma_{\Delta }\\ = & I_{n_{x}}-\Sigma_{\Delta }Q_{X|Y}^{-1}=H\;\;\;\;\mathrm{since}\;Q_{X|Y}\;\mathrm{and}\;\Sigma_{\Delta }\;\mathrm{commute}. \end{array}$$

By the definition of $Q_{W}$ given in Theorem 4.(a), we have

(A44) $$\begin{array}{c} Q_{W}=\Sigma_{\Delta }H^{T}=Q_{W}^{T}=H\Sigma_{\Delta }. \end{array}$$

Substituting (A43) into (A44), then

(A45) $$\begin{array}{c} Q_{W}=\Sigma_{\Delta }H. \end{array}$$

Hence, $\left\{\Sigma_{\Delta },\Sigma_{X|Y},H,Q_{W}\right\}$ are all elements of $S_{+}^{p\times p}$ having a spectral decomposition with respect to the same unitary matrix $UU^{T}=I_{n_{x}}$.
